# Transformer‐Based Preoperative Invasiveness Identification and Postoperative Recurrence Risk Stratification in Stage T1N0M0 Lung Adenocarcinoma: A Multi‐Center Cohort Study

**DOI:** 10.1002/mco2.71055

**Published:** 2026-10-11

**Authors:** Xueyun Tan, Hui Xia, Delong Li, Meifang Wang, Na Zhan, Yan Huang, Mengyuan Liang, Pei Ma, Xiao Wang, Yaqi Cao, Linpeng Shi, Liang Zhang, Xiaoling Liu, Lian Yang, Quan Kong, Sufei Wang, Yang Jin

**Affiliations:** ^1^ Department of Respiratory and Critical Care Medicine Hubei Province Clinical Research Center for Major Respiratory Disease Union Hospital Tongji Medical College Huazhong University of Science and Technology Wuhan China; ^2^ Department of Respiratory and Critical Care Medicine Zhongnan Hospital of Wuhan University Wuhan China; ^3^ Department of Respiratory and Critical Care Medicine China‐Japan Friendship Hospital Beijing China; ^4^ Hubei Key Laboratory of Optical Information and Pattern Recognition School of Optical Information and Energy Engineering Wuhan Institute of Technology Wuhan China; ^5^ Department of Respiratory and Critical Care Medicine Shiyan Key Laboratory of Major Chronic Respiratory Disease, Taihe Hospital Hubei University of Medicine Shiyan China; ^6^ Department of Pathology Renmin Hospital of Wuhan University Wuhan China; ^7^ Department of Respiratory and Critical Care Medicine, Xiangyang Central Hospital Affiliated Hospital of Hubei University of Arts and Science Xiangyang China; ^8^ Department of Respiratory and Critical Care Medicine, Henan Provincial People's Hospital Zhengzhou University Zhengzhou China; ^9^ Department of Respirology Guangzhou Institute of Respiratory Health The First Affiliated Hospital of Guangzhou Medical University State Key Laboratory of Respiratory Diseases Guangzhou China; ^10^ Hubei Province Key Laboratory of Biological Targeted Therapy, MOE Key Laboratory of Biological Targeted Therapy, Union Hospital, Tongji Medical College Huazhong University of Science and Technology Wuhan China; ^11^ Hubei Province Engineering Research Center for Tumor‐Targeted Biochemotherapy, Union Hospital, Tongji Medical College Huazhong University of Science and Technology Wuhan China; ^12^ Department of Radiology Renmin Hospital of Wuhan University Wuhan China; ^13^ Institute of Research and Clinical Innovations Neusoft Medical Systems Co. Ltd Wuhan China; ^14^ Department of Radiology Union Hospital, Tongji Medical College Huazhong University of Science and Technology Wuhan China; ^15^ Hubei Key Laboratory of Optical Information and Pattern Recognition Wuhan Institute of Technology Wuhan China

**Keywords:** lung adenocarcinoma, invasiveness, recurrence risk, transformer, multimodal information

## Abstract

Surgery is the primary curative treatment for early‐stage lung adenocarcinoma (LUAD), with accurate identification of tumor invasiveness critical for determining pulmonary resection extent. Due to significant variability in postoperative recurrence among early‐stage LUAD patients with aggressive tumors, a more effective risk stratification system is urgently needed to improve prognosis. This study developed the T1LUT system, a transformer‐based tool for T1N0M0 LUAD management, covering preoperative tumor invasiveness identification and postoperative recurrence risk stratification. Preoperatively, a 3D Vision Transformer (ViT) with extra learnable tokens analyzes CT images. It outperforms 3D ViT with only CLS tokens, 2D ViT, and 3D ResNet across three independent test centers (Center D: 0.896 vs. 0.892, 0.879, 0.836; Center E: 0.906 vs. 0.837, 0.837, 0.835; Center F: 0.884 vs. 0.884, 0.881, 0.861), achieving higher mean AUROC to accurately assess invasiveness and guide surgical resection extent. Postoperatively, the multimodal IAC‐PR model integrates CT images, reports, pathological data, and clinical/demographic information. It stratifies invasive adenocarcinoma (IAC) patients into low‐, medium‐, or high‐risk groups with an AUROC of 0.807 in testing—significantly better than single/partial‐modal models. Validated via large‐scale multicenter research, this system overcomes traditional assessment limitations, offering a robust tool for precise early LUAD treatment and improved prognosis.

## Graphical abstract

This study developed a transformer‐based decision‐making system (the T1LUT system) that operates at two critical timepoints: preoperative identification of tumor invasiveness and postoperative stratification of recurrence risk in stage T1N0M0 LUAD. For preoperative invasiveness identification, the T1LUT system employs a Vision Transformer (ViT)‐based model using preoperative CT images. For postoperative management, the T1LUT system further stratifies recurrence risk in patients with invasive adenocarcinoma (IAC) using a multimodal IAC‐PR model. This model integrates CT images, CT reports, pathologic reports, and clinical and demographic data within a transformer framework. This system provides a reliable, noninvasive tool with significant potential to improve clinical decision‐making and patient outcomes.

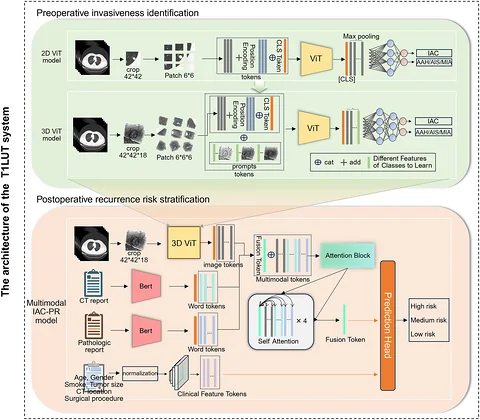

## 1 Introduction

The management of early‐stage lung cancer, notably small peripheral non‐small‐cell lung cancer (NSCLC), increasingly hinges on selecting the right surgical procedure. This is underscored by the current pathologic classification of lung adenocarcinoma (LUAD), which since 2011 has distinguished atypical adenomatous hyperplasia (AAH) and adenocarcinoma in situ (AIS) as preinvasive lesions from minimally invasive adenocarcinoma (MIA) and invasive adenocarcinoma (IAC) [1]. Determining the optimal resection strategy is essential for achieving the best possible results for patients [2]. Although lobectomy remains the standard procedure for resectable NSCLC, several studies have demonstrated that limited resection—including segmentectomy and wedge resection—can achieve curative effectiveness non‐inferior to lobectomy for small‐sized NSCLC with less aggressive behaviors, while better preserving pulmonary function [2, 3, 4]. Accurate identification of tumor invasiveness is essential for determining the extent of pulmonary resection [5], and many studies have reported that radiological features of pulmonary nodules, such as spiculation and solid component size, are strongly associated with malignancy risk [6, 7]. Therefore, CT images are the most commonly used data for predicting the invasiveness of LUAD, either through feature extraction or direct model input [8, 9]. However, the CT image preprocessing pipelines of current studies are complex and lack standardization, hindering efficient data handling. Additionally, many studies restrict their scope to specific nodule types, such as pure ground‐glass or subsolid nodules [9, 10]. This narrow focus not only limits the general applicability of the developed models but also increases the workload of data curation.

Following the identification of invasiveness and subsequent curative resection, assessing the risk of postoperative recurrence becomes a critical clinical consideration. Despite the effectiveness of curative‐intent surgery, 20%–40% of patients with stage I NSCLC experience postoperative recurrence, and the 5‐year post‐recurrence survival rate ranges from 15% to 16.6%, representing a significant clinical challenge [11, 12, 13, 14]. To assist in indicating the risk of recurrence, in 2020, the International Association for the Study of Lung Cancer (IASLC) proposed a novel grading system that classifies invasive LUAD into three histologic differentiated subtypes based on the predominant histologic pattern and the proportion of high‐grade histologic components [15]. External validations of this novel grading system have been conducted in several studies. Rokutan‐Kurata et al. reported that the IASLC grading system effectively predicted recurrence and mortality in invasive LUAD [16]. Kagimoto et al. found significant differences in recurrence‐free survival (RFS) among patients with pathologic stage 0 or I LUAD when stratified by the IASLC grading system [17]. However, only 30% of patients with early‐stage LUAD classified as grade 3 experience postoperative recurrence [18]. Given the heterogeneous outcomes in postoperative recurrence among early‐stage LUAD patients, there is a need for a more effective recurrence risk stratification system to improve prognosis. Therefore, a new postoperative recurrence risk stratification was proposed in this study. Patients with IAC who had undergone surgery and were followed for more than 3 years were stratified into three recurrence risk categories‐low, medium, and high‐based on a combination of the IASLC grading system and follow‐up information. The detailed definition was illustrated in Section 4.

Based on the above considerations, this study aims to develop a transformer‐based decision‐making system (the T1LUT system) that operates at two critical timepoints‐preoperative identification of tumor invasiveness and postoperative stratification of recurrence risk‐to provide continuous, personalized management for patients with stage T1N0M0 LUAD. The core architecture of this system is the Vision Transformer (ViT), which has attracted broad interest in medical image analysis due to its strengths in capturing long‐range spatial correlations and handling variable‐length inputs [19, 20]. Transformer‐based diagnostic models have also shown remarkable ability in integrating multimodal input data. Zhou et al. reported a unified multimodal transformer that integrated chest x‐ray images and multiple types of clinical text data to diagnose pulmonary diseases and predict adverse outcomes in COVID‐19 patients [21]. In our study, we incorporated additional learnable tokens across the T1LUT system to capture more diverse and discriminative features, thereby improving model performance. This token‐level fusion approach also enables a seamless transition and unified modeling from unimodal to multimodal learning [22].

For the preoperative component of the T1LUT system to identify invasiveness, we developed a 3D ViT model using preoperative CT images. The proposed 3D ViT model, enhanced with extra learnable tokens, was evaluated against three baseline models: a 3D ViT with only CLS tokens, a 2D ViT, and a 3D ResNet. It achieved the highest area under the receiver operating characteristic curve (AUROC) across all three independent testing sets (Center D: 0.896; Center E: 0.906; Center F: 0.884). For the postoperative phase, we constructed a multimodal IAC‐PR model to predict recurrence risk in patients with IAC confirmed by formalin‐fixed paraffin‐embedded (FFPE) section analysis. This model integrated preoperative CT images, CT reports, pathologic reports, and clinical‐demographic data. In the testing set (Center D), the multimodal IAC‐PR model attained an AUROC of 0.807, outperforming all other input modalities. Both models within the T1LUT system were rigorously cross‐validated and tested on large‐scale, multi‐center cohorts, demonstrating superior accuracy and robustness. Digital interventions have shown substantial effects on health management [23], with especially important implications for precision diagnosis and treatment in oncology.

## 2 Results

### 2.1 Patient Characteristics for Preoperative Invasiveness Identification

Upon the application of the inclusion and exclusion criteria, 5031 patients from three hospitals were enrolled to perform cross‐validation of the models for invasiveness identification, and 1447 patients from three additional hospitals were included as independent testing sets. For each patient, demographic information (age, sex, and smoking history), CT features of the target pulmonary nodule (size and location), pathologic type, and surgical procedure were collected. The clinical characteristics of patients from each center are summarized in Table 1. Across all six centers, females comprised the majority (> 60%), and most patients had no smoking history (> 75%). The right upper lobe was the most common location for pulmonary nodules, while the right middle lobe was the least common. Lobectomy was the most frequently performed surgical procedure in Center A (57.8%), Center B (42.9%), Center C (39.4%), Center D (47.3%), and Center E (83.8%). In these five centers, IAC accounted for the largest proportion of cases (67.6%, 69.7%, 60.0%, 66.5%, and 48.9%, respectively). By contrast, in Center F, wedge resection was the most common surgical approach (43.9%), and MIA represented the largest histologic subtype (46.8%).

**TABLE 1:** Clinical characteristics of patients in the datasets enrolled for preoperative invasiveness identification.

Characteristics	Training set			Testing sets		
	Center A (*n* = 2358)	Center B (*n* = 1502)	Center C (*n* = 1171)	Center D (*n* = 884)	Center E (*n* = 358)	Center F (*n* = 205)
Age (years, IQR)	58 (50–65)	57 (51–64)	59 (53–66)	58 (51–65)	57 (48–66)	53 (44–62)
Gender						
Male	888 (37.7%)	571 (38.0%)	391 (33.4%)	308 (34.8%)	116 (32.4%)	79 (38.5%)
Female	1470 (62.3%)	931 (62.0%)	780 (66.6%)	576 (65.2%)	242 (67.6%)	126 (61.5%)
Smoking history						
No	2007 (85.1%)	1156 (77.0%)	977 (83.4%)	741 (83.8%)	296 (82.7%)	190 (92.7%)
Yes	351 (14.9%)	346 (23.0%)	194 (16.6%)	143 (16.2%)	62 (17.3%)	15 (7.3%)
Tumor size on CT (mm, IQR)	14 (10–19)	14 (11–20)	14 (11–19)	14 (10–18)	12 (8–17)	10 (8–14)
CT location						
Right upper lobe	846 (35.9%)	515 (34.3%)	409 (34.9%)	328 (37.1%)	112 (31.3%)	64 (31.2%)
Right middle lobe	128 (5.4%)	123 (8.2%)	72 (6.2%)	61 (6.9%)	16 (4.5%)	13 (6.3%)
Right lower lobe	419 (17.7%)	263 (17.5%)	205 (17.5%)	144 (16.3%)	65 (18.2%)	37 (18.1%)
Left upper lobe	624 (26.5%)	390 (26.0%)	309 (26.4%)	229 (25.9%)	106 (29.5%)	51 (24.9%)
Left lower lobe	341 (14.5%)	211 (14.0%)	176 (15.0%)	122 (13.8%)	59 (16.5%)	40 (19.5%)
Pathology						
AAH	8 (0.3%)	2 (0.1%)	27 (2.3%)	3 (0.3%)	1 (0.3%)	3 (1.5%)
AIS	247 (10.5%)	148 (9.9%)	156 (13.3%)	134 (15.2%)	29 (8.1%)	31 (15.1%)
MIA	509 (21.6%)	305 (20.3%)	285 (24.4%)	159 (18.0%)	153 (42.7%)	96 (46.8%)
IAC	1594 (67.6%)	1047 (69.7%)	703 (60.0%)	588 (66.5%)	175 (48.9%)	75 (36.6%)
Surgical procedure						
Lobectomy	1364 (57.8%)	645 (42.9%)	461 (39.4%)	418 (47.3%)	300 (83.8%)	62 (30.2%)
Segmentectomy	787 (33.4%)	606 (40.4%)	444 (37.9%)	343 (38.8%)	56 (15.6%)	53 (25.9%)
Wedge resection	207 (8.8%)	251 (16.7%)	266 (22.7%)	123 (13.9%)	2 (0.6%)	90 (43.9%)

*Note*: Data are presented as number (percentage, %), with the exception of age and tumor size on CT (median [IQR]).

Abbreviations: AAH, atypical adenomatous hyperplasia; AIS, adenocarcinoma in situ; CT, computed tomography; IAC, invasive adenocarcinoma; IQR, interquartile range; MIA, minimally invasive adenocarcinoma.

### 2.2 3D ViT Model Can Accurately Identify the Invasiveness in Patients With Clinical Stage T1N0M0 LUAD

As the first component of the T1LUT system, CT images were used to construct models based on the ViT architecture to identify tumor invasiveness preoperatively in patients with clinical stage T1N0M0 LUAD. In fivefold cross‐validation on the training set (Center A, B, and C), the 3D ViT model achieved a higher mean AUROC of 0.888 (95%CI: 0.883–0.893) (Figure 1B) compared with the 2D ViT model (0.860, 95% CI: 0.855–0.865) (Figure 1A). Across all three independent testing sets, the 3D ViT model demonstrated consistently better performance than the 2D ViT model. Specifically, it achieved higher mean AUROC values (Center D: 0.896 vs. 0.879; Center E: 0.906 vs. 0.837; Center F: 0.884 vs. 0.881) and higher mean accuracy (Center D: 0.807 vs. 0.751; Center E: 0.771 vs. 0.737; Center F: 0.839 vs. 0.819) (Table S1). The performance of the 3D ViT model without additional learnable tokens (3D ViT [CLS only]) is presented in Figure S1 and Table S1, showing that the inclusion of learnable tokens improved the model's performance.

**FIGURE 1:**
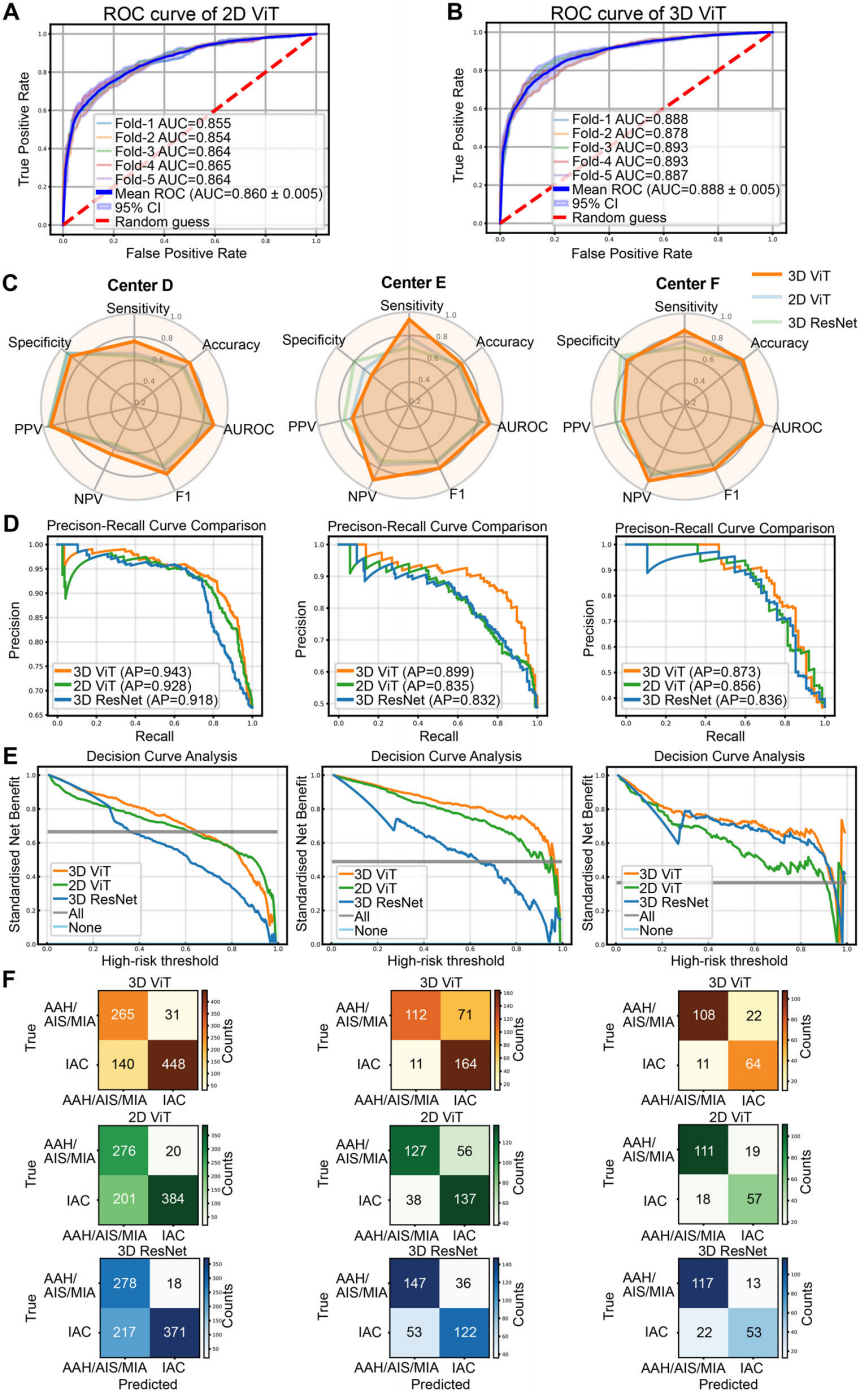
A fivefold cross‐validation was conducted during the training of the 2D ViT model (A) and 3D ViT model (B), respectively, for invasiveness identification, and the AUROCs were plotted along with the 95% CI region. (C) Radar maps of the seven predictive performance indexes (AUROC, accuracy, sensitivity, specificity, PPV, NPV, and F1 score) for 3D ViT model, 2D ViT model, and 3D ResNet model in three independent testing sets. (D) Comparison of precision‐recall curves among 3D ViT model, 2D ViT model, and 3D ResNet model in three independent testing sets. (E) Comparison of decision curve analysis among 3D ViT model, 2D ViT model, and 3D ResNet model in three independent testing sets. (F) Comparison of confusion matrices for invasiveness prediction among 3D ViT model, 2D ViT model, and 3D ResNet model in three independent testing sets. AUROC, the area under receiver operating characteristic curve; NPV, negative predictive value; PPV, positive predictive value.

Residual networks (ResNet), first introduced by He et al. in 2016, are considered state‐of‐the‐art convolutional neural networks (CNNs) [24]. Therefore, we further compared the 2D ViT and 3D ViT models with a well‐trained 3D ResNet model developed by Chen et al. and later applied by Wang et al. in pulmonary nodule risk stratification and precision management studies [25, 26]. As shown in Figure 1C, the 3D ViT model outperformed both the 2D ViT and 3D ResNet models in AUROC, accuracy, sensitivity, negative predictive value (NPV), and F1 score across the three independent testing sets. Similarly, the area under precision‐recall curve (AUPRC) of the 3D ViT model was superior to that of the other two models (Center D: 0.943 vs. 0.928 vs. 0.918; Center E: 0.899 vs. 0.835 vs. 0.832; Center F: 0.873 vs. 0.856 vs. 0.836) (Figure 1D). Decision curve analysis further demonstrated that the 3D ViT model provided a higher net clinical benefit than the 2D ViT and 3D ResNet models. Specifically, this advantage was observed when the decision threshold probability was between 0.15–0.40 or 0.60–0.90 in Center D, 0.40–0.90 in Center E, and 0.60–0.90 in Center F (Figure 1E). Consistently, confusion matrices indicated that the 3D ViT model predictions aligned more closely with actual invasiveness classifications than those of the 2D ViT or 3D ResNet models (Figure 1F).

### 2.3 Comparison of the Heatmaps Among Different Invasiveness Type and Subgroup Analysis

Figure S2 presents CT images and corresponding heatmaps with predicted invasiveness types for four representative cases. The activation heatmaps highlight the relative importance of different lesion regions, visually suggesting that imaging features at the nodule margins represent the most critical predictors of invasiveness. For relatively larger nodules, imaging characteristics from the central regions also contributed substantially to invasiveness classification. Figure S3 displays CT images of pulmonary nodules with typical malignant radiological features (pleural traction, lobular sign, spiculation, vascular convergence) and corresponding attention heatmaps from the CLS token and an extra learnable token. The CLS token consistently focuses on the nodule parenchyma for reliable lesion localization, while the extra learnable token effectively attends to the surrounding diagnostic radiological signs. This complementary attention pattern enables the model to simultaneously capture lesion location and malignant morphological features, enhancing the performance and interpretability of lung nodule diagnosis.

A subgroup analysis based on tumor size (< 10 mm, 10–20 mm, and 20–30 mm) was conducted to further evaluate the predictive performance of the 3D ViT model in the testing sets (Table S2). The model achieved the highest AUROC in the 10–20 mm group (0.834, 95% CI: 0.806–0.862) and the highest accuracy in the 20–30 group (0.896, 95% CI: 0.847–0.930). The distribution of invasiveness types across the three subgroups was as follows: < 10 mm group: 16.3% IAC, 50.0% MIA, 33.7% AAH/AIS; 10–20 mm group: 69.6% IAC, 23.5% MIA, 6.9% AAH/AIS; 20–30 mm group: 98.6% IAC, 1.4% MIA, 0% AAH/AIS. These results indicate that the proportion of IAC increases sharply with tumor size, while the frequency of preinvasive or minimally invasive lesions decreases accordingly.

### 2.4 Patient Characteristics for Postoperative Recurrence Risk Stratification

After excluding patients lost to follow‐up, 595 IAC cases from Center A, 380 from Center B, and 164 from Center C were included in the training set for fivefold cross‐validation to train the models for postoperative recurrence risk stratification. Additional 262 IAC cases from Center D were used as the independent testing set. As shown in Table 2, females comprised the majority of patients in all four centers (58.7% in Center A, 60.8% in Center B, 61.0% in Center C, and 62.6% in Center D). According to the IASLC grading system, grade 2 (moderately differentiated) cases were the most common in all four centers (73.5% in Center A, 46.3% in Center B, 78.0% in Center C, and 47.3% in Center D). The proportion of grade 3 (poorly differentiated) cases exceeded that of grade 1 (well differentiated) in Center A (17.8% vs. 8.7%), Center C (15.3% vs. 6.7%), and Center D (37.4% vs. 15.3%). By contrast, in Center B, grade 1 cases were more frequent than grade 3 cases (41.6% vs. 12.1%). The proportions of IAC patients who experienced recurrence within 3 years after surgery were 6.4% in Center A, 10.5% in Center B, 6.7% in Center C, and 9.9% in Center D. Based on the recurrence risk stratification criteria, patients in each center were categorized into high‐, medium‐, and low‐risk groups.

**TABLE 2:** Clinical Characteristics of Patients in the datasets enrolled for postoperative recurrence risk stratification.

Characteristics	Training set			Testing set (*n* = 262)
	Center A (*n* = 595)	Center B (*n* = 380)	Center C (*n* = 164)	Center D (*n* = 262)
Age (years, IQR)	58 (50–64)	58 (52–65)	60 (54–66)	60 (53–66)
Gender				
Male	246 (41.3%)	149 (39.2%)	64 (39.0%)	98 (37.4%)
Female	349 (58.7%)	231 (60.8%)	100 (61.0%)	164 (62.6%)
Smoking history				
No	483 (81.2%)	278 (73.2%)	128 (78.0%)	204 (77.9%)
Yes	112 (18.8%)	102 (26.8%)	36 (22.0%)	58 (22.1%)
Tumor size on CT (mm, IQR)	16 (13–20)	17 (14–23)	17 (15–31)	17 (14–21)
CT Location				
Right upper lobe	219 (36.8%)	146 (38.4%)	59 (36.0%)	59 (22.5%)
Right middle lobe	23 (3.9%)	34 (8.9%)	11 (6.7%)	36 (13.7%)
Right lower lobe	122 (20.5%)	61 (16.1%)	24 (14.6%)	104 (39.7%)
Left upper lobe	143 (24.0%)	85 (22.4%)	45 (27.4%)	17 (6.5%)
Left lower lobe	88 (14.8%)	54 (14.2%)	25 (15.2%)	46 (17.6%)
IASLC grading system (differentiated degree in pathology)				
Grade 3 (poorly differentiated)	106 (17.8%)	46 (12.1%)	25 (15.3%)	98 (37.4%)
Grade 2 (moderately differentiated)	437 (73.5%)	176 (46.3%)	128 (78.0%)	124 (47.3%)
Grade 1 (well differentiated)	52 (8.7%)	158 (41.6%)	11 (6.7%)	40 (15.3%)
Surgical procedure				
Lobectomy	485 (81.5%)	261 (68.7%)	119 (72.5%)	151 (57.6%)
Segmentectomy	75 (12.6%)	107 (28.1%)	38 (23.2%)	88 (33.6%)
Wedge resection	35 (5.9%)	12 (3.2%)	7 (4.3%)	23 (8.8%)
Recurrence within 3 years after operation				
Yes	38 (6.4%)	40 (10.5%)	11 (6.7%)	26 (9.9%)
No	557 (93.6%)	340 (89.5%)	153 (93.3%)	236 (90.1%)
Recurrence risk stratification				
High risk	38 (6.4%)	40 (10.5%)	11 (6.7%)	26 (9.9%)
Medium risk	96 (16.1%)	34 (9.0%)	23 (14.0%)	36 (13.8%)
Low risk	461 (77.5%)	306 (80.5%)	130 (79.3%)	200 (76.3%)

*Note*: Data are presented as number (percentage, %), with the exception of age and tumor size on CT (median [IQR]).

Abbreviations: CT, computed tomography; IQR, interquartile range.

### 2.5 Multimodal IAC‐PR Model Outperforms Other Models With Different Input Modalities on Predicting the Postoperative Recurrence Risk

For the second part of the T1LUT system, multimodal information—including CT images, CT reports, pathology reports, and clinical and demographic data—was integrated to develop the multimodal IAC‐PR model for stratifying postoperative recurrence risk in IAC patients. Using fivefold cross‐validation, the model achieved mean AUROCs of 0.699 (95% CI: 0.645–0.753), 0.906 (95% CI: 0.876–0.936), and 0.856 (95% CI: 0.821–0.891) for identifying high‐, medium‐, and low‐risk groups, respectively. The overall micro‐averaged AUROC and macro‐averaged AUROC were 0.903 (95% CI: 0.888–0.918) and 0.820 (95% CI: 0.788–0.852), respectively (Figure 2A).

**FIGURE 2:**
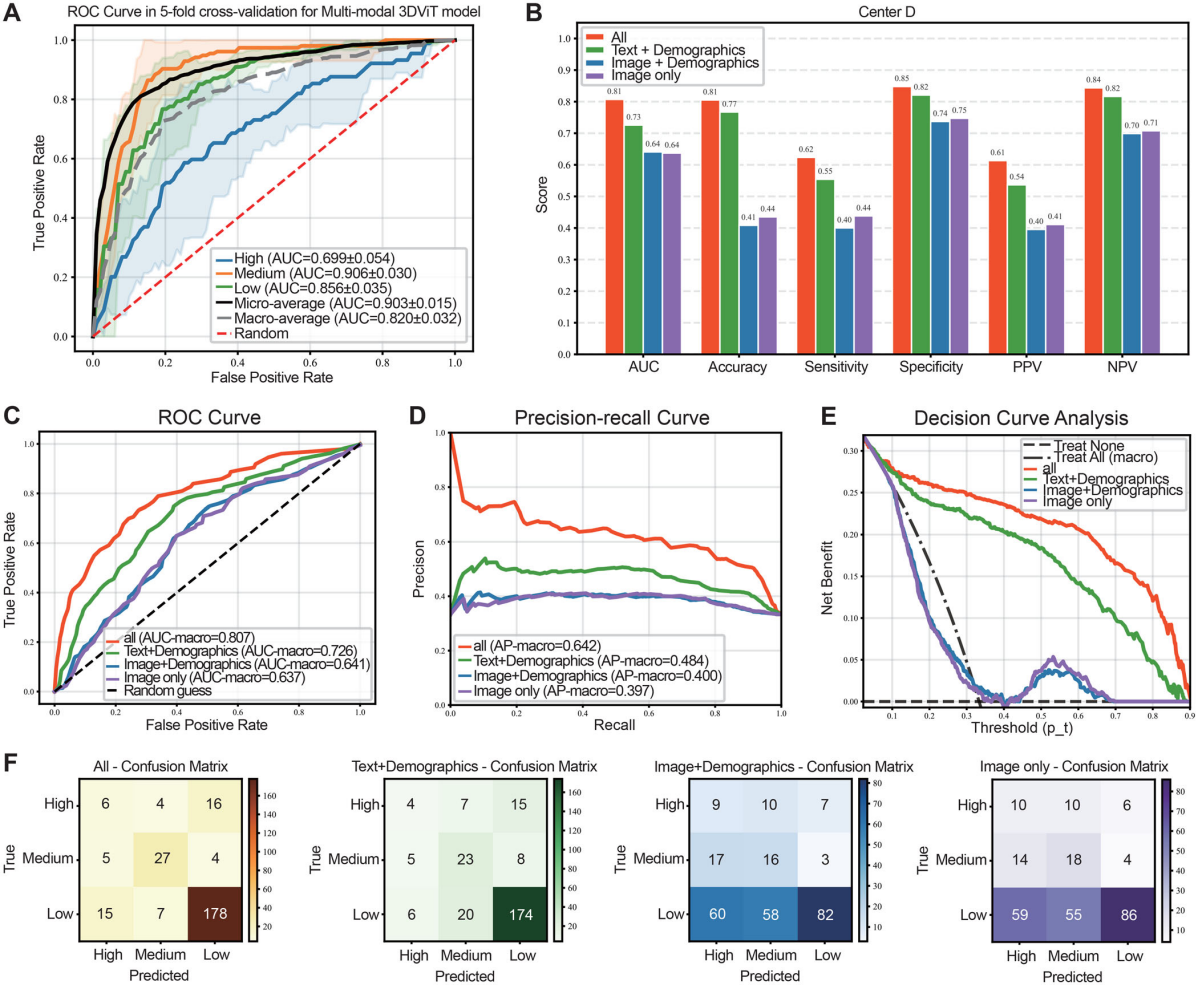
Multimodal IAC‐PR model's performance in fivefold cross‐validation and comparison of predictive performance among models with different input modalities in Center D testing set for postoperative stratification of recurrence risk. “All” means multimodal IAC‐PR model. (A) ROC curve of fivefold cross‐validation during training for multimodal IAC‐PR model to predict the postoperative recurrence risk with AUCs of high risk, medium risk, low risk, micro‐average, and macro‐average, all showing in the plot. (B) Comparison of macro‐averaged six performance indexes (AUROC, accuracy, sensitivity, specificity, PPV, and NPV) among models with different input modalities. (C) Comparison of ROC curves among models with different input modalities in Center D testing set. (D) Comparison of precision‐recall curves among models with different input modalities in Center D testing set. (E) Comparison of decision curve analysis among models with different input modalities in Center D testing set. (F) Comparison of confusion matrices for postoperative recurrence risk stratification among models with different input modalities in Center D testing set. Abbreviations: AUROC, the area under receiver operating characteristic curve; NPV, negative predictive value; PPV, positive predictive value.

We further compared the performance of models with different input modalities in the testing set (Center D). In the independent testing set (Center D), the multimodal IAC‐PR model demonstrated the highest mean accuracy (0.805, 95% CI: 0.753–0.849) compared with models using individual or partial modalities (0.435, 95% CI: 0.376–0.496; 0.408, 95% CI: 0.351–0.469; and 0.767, 95% CI: 0.712–0.814) (Table S3). Clinical and demographic information contributed minimally to performance, as indicated by the small AUROC improvement from the image‐only model (AUROC = 0.637, 95% CI: 0.570–0.701) to the image + demographics model (AUROC = 0.641, 95% CI: 0.573–0.710; ΔAUROC = 0.004). By contrast, textual information extracted from CT and pathology reports had a much greater impact. Adding textual data increased AUROC from 0.641 (95% CI: 0.573–0.710) to 0.807 (95% CI: 0.750–0.860; ΔAUROC = 0.166). Notably, textual features alone provided a larger performance gain than CT image features alone (ΔAUROC = 0.081, AUROC = 0.726, 95% CI: 0.662–0.786 to AUROC = 0.807, 95% CI: 0.750–0.860) (Figure 2B,C). Precision‐recall curve analysis (Figure 2D), decision curve analysis (Figure 2E), and confusion matrices (Figure 2F) all demonstrated that the multimodal IAC‐PR model outperformed models trained with individual or partial modalities.

### 2.6 Importance Evaluation of the Textual Information and the Clinical and Demographic Information and the Subgroup Analysis for Grade 2 Patients

Among the top 10 important keywords extracted from CT and pathology reports, “solid” had the strongest impact in both imaging and pathology domains (Figure S4). In addition to histologic‐pattern‐related keywords from pathology reports, morphological descriptors from CT reports—such as “ground‐glass opacity” and “spiculated margin”—also made substantial contributions to model performance (Figure S4).

We further examined differences in clinical characteristics across high‐, medium‐, and low‐risk groups in the cross‐validation and testing sets for the task of postoperative recurrence risk stratification (Table 3). In the training set, gender (*p* = 0.005), smoking history (*p* = 0.006), tumor size on CT (*p* < 0.001), CT location (*p* = 0.002), and surgical procedure (*p* < 0.001) differed significantly among the three groups. In the testing set, gender (*p* = 0.002) and tumor size on CT (*p* < 0.001) showed significant differences. Within the high‐risk group, grade 2 tumors accounted for the largest proportion in both the cross‐validation (64.0%) and testing (50.0%) sets (Table 3).

**TABLE 3:** Comparison of clinical characteristics among IAC patients with different postoperative stratification of recurrence risk.

	Training set			*p* value	Testing set (*n* = 262)			
Characteristics	High risk (*n* = 89)	Medium risk (*n* = 153)	Low risk (*n* = 897)		High risk (*n* = 26)	Medium risk (*n* = 36)	Low risk (*n* = 200)	*p* value
Age (years, IQR)	59 (54–65)	57 (50–65)	58 (52–65)	0.27	63.5 (54–69)	58.5 (53–65.5)	59 (52–66)	0.21
Gender								
Male	46 (51.7%)	73 (47.7%)	340 (37.9%)	0.005	15 (57.7%)	20 (55.6%)	63 (31.5%)	0.002
Female	43 (48.3%)	80 (52.3%)	557 (62.1%)		11 (42.3%)	16 (44.4%)	137 (68.5%)	
Smoking history								
No	60 (67.4%)	111 (72.5%)	717 (79.9%)	0.006	16 (61.5%)	26 (72.2%)	162 (81.0%)	0.054
Yes	29 (32.6%)	42 (27.5%)	180 (20.1%)		10 (38.5%)	10 (27.8%)	38 (19.0%)	
Tumor size on CT (mm, IQR)	18.5 (14–23)	17 (13–22)	16 (12–21)	< 0.001	15.5 (14–20)	17 (13–20)	13 (10–16.5)	< 0.001
CT location								
Right upper lobe	28 (31.5%)	42 (27.4%)	352 (39.2%)	0.002	10 (38.5%)	14 (38.9%)	81 (40.5%)	0.76
Right middle lobe	4 (4.5%)	11 (7.2%)	54 (6.0%)		3 (11.5%)	3 (8.3%)	11 (5.5%)	
Right lower lobe	13 (14.6%)	32 (20.9%)	161 (18.0%)		3 (11.5%)	9 (25.0%)	33 (16.5%)	
Left upper lobe	26 (29.2%)	31 (20.3%)	219 (24.4%)		7 (26.9%)	5 (13.9%)	47 (23.5%)	
Left lower lobe	18 (20.2%)	37 (24.2%)	111 (12.4%)		3 (11.5%)	5 (13.9%)	28 (14.0%)	
Differentiated degree in pathology								
Grade 3 (poorly differentiated)	24 (27.0%)	153 (100.0%)	0	< 0.001	4 (15.4%)	36 (100.0%)	0	< 0.001
Grade 2 (moderately differentiated)	57 (64.0%)	0	681 (75.9%)		13 (50.0%)	0	111 (55.5%)	
Grade 1 (well differentiated)	8 (9.0%)	0	216 (24.1%)		9 (34.6%)	0	89 (44.5%)	
Surgical procedure								
Lobectomy	70 (78.7%)	137 (89.5%)	658 (73.4%)	< 0.001	13 (50.0%)	25 (69.4%)	113 (56.5%)	0.24
Segmentectomy	14 (15.7%)	7 (4.6%)	199 (22.2%)		12 (46.2%)	7 (19.4%)	69 (34.5%)	
Wedge resection	5 (5.6%)	9 (5.9%)	40 (4.5%)		1 (3.8%)	4 (11.1%)	18 (9.0%)	

*Note*: Data are presented as number (percentage, %), with the exception of age and tumor size on CT (median [IQR]).

Abbreviations: CT, computed tomography; IQR, interquartile range.

Figure S5 shows the performance of the multimodal IAC‐PR model for grade 2 patients in the training (*n* = 738) and testing (*n* = 124) sets. In the training set, the distribution of risk categories was 57 (7.7%) high‐risk, 0 (0%) medium‐risk, and 681 (92.3%) low‐risk cases. The model achieved an AUROC of 0.842 (95% CI: 0.790–0.887) and an accuracy of 0.780 (95% CI: 0.749–0.809). In the testing set, the risk distribution was 13 (10.5%) high‐risk, 0 (0%) medium‐risk, and 111 (89.5%) low‐risk cases. The model attained an AUROC of 0.714 (95% CI: 0.509–0.873) and an accuracy of 0.839 (95% CI: 0.774–0.903). The confusion matrices further illustrate the model's classification performance. The model exhibited high specificity and NPV across cohorts, with reliable generalization to the testing set.

## 3 Discussion

As the leading cause of cancer‐related mortality, lung cancer imposes a substantial global economic burden. In recent years, the precision management of early‐stage lung cancer has attracted increasing attention. To address critical decision points in the clinical pathway of T1N0M0 LUAD, we propose the T1LUT system. At the preoperative stage, it incorporates a 3D ViT model for non‐invasive invasiveness assessment from CT scans, directly informing surgical strategy. Following surgery and pathologic confirmation of IAC, the system leverages a multimodal IAC‐PR model—fusing imaging, radiology/pathology reports, and clinical data—to provide personalized recurrence risk stratification, thereby guiding post‐operative follow‐up and adjuvant therapy considerations. Patients were stratified into three risk levels—low, medium, and high—based on the IASLC grading system combined with follow‐up information.

For preoperative invasiveness identification, the 3D ViT model consistently demonstrated superior performance and robustness across cross‐validation and independent testing datasets. Despite relying solely on chest CT images—without incorporating CT reports or clinical and demographic data—the model exhibited exceptional predictive performance. In clinical practice, chest CT is routinely included in health examinations for asymptomatic screening [27]. Thus, the 3D ViT model can predict invasiveness without waiting for radiologist evaluations or additional clinical data, making it easily adaptable to the workflow of CT examinations. In addition, the design of the learnable token was pivotal: for the 3D ViT model, the extra learnable token extracted fine‐grained semantic features, improving the model's ability to capture local lesion details without altering the ViT backbone. For postoperative recurrence risk stratification, this mechanism was extended into a fusion token, enabling integration of multimodal data. Through the self‐attention mechanism, it established high‐order correlations across modalities, explicitly aggregating and interacting with global information [28]. This additional learnable token thus serves as both a conceptual link between the two tasks and a key module driving fine‐grained feature extraction, cross‐modal fusion, and cross‐domain generalization. This token‐level fusion approach offers several advantages. The fusion token functions as a learnable query that explicitly aggregated information from all modalities, thereby avoiding information loss often associated with simple averaging or pooling strategies. Moreover, this method is highly flexible and can be seamlessly extended to incorporate additional modalities without modifying the fusion mechanism itself. Collectively, it offers a novel strategy for constructing a unified, efficient medical multimodal analysis framework.

Predicting recurrence after complete resection of early‐stage NSCLC has been a persistent challenge, particularly for node‐negative cases, in which recurrence rates range from 13% to 23% [29, 30]. Numerous studies have sought to develop prognostic biomarkers for recurrence. Jones et al. developed a PRecur prediction model using 14 clinicopathologic and genomic variables for RFS risk stratification in patients with completely resected stages I to III LUAD [12]. Although it outperformed TNM‐based models, its concordance probability estimate was not high (0.70–0.73). Similarly, Chen et al. reported a model integrating 16 genomic and clinical features, which achieved an AUC of 0.84 to discriminate high risk and low risk of recurrence in stage I NSCLC [14]. Although these models revealed some molecular mechanism related to tumor recurrence in early‐stage NSCLC, they were less practical as many unconventional indicators require invasive detective technique, and the generalizability of these models was not confirmed.

Since the introduction of the IASLC grading system, multiple studies have validated its prognostic and predictive value for IAC, and its clinical utility has been widely recognized, albeit with room for improvement [15, 17]. Hou et al. further confirmed that stage I invasive LUAD patients classified as grade 3 had significantly worse RFS and overall survival (OS) compared with those in grades 1 and 2 [31]. However, it is noteworthy that only ∼30% of early‐stage LUAD patients classified as grade 3 actually experience postoperative recurrence [18]. This highlights the pressing need for additional clinical and biological parameters to complement the IASLC grading system. Other indicators, such as solid component size on CT, lymphovascular invasion, and surgical approach, have also been shown to correlate with postoperative recurrence risk [4, 32, 33]. Building upon this evidence, our study proposed a more refined recurrence risk stratification strategy by combining the IASLC grading system with follow‐up data. These clinicopathologic variables, together with imaging and textual data, constituted the multimodal dataset used to train the IAC‐PR model. Consistent with prior findings, solid features and tumor size emerged as highly influential contributors to risk stratification in our models. Importantly, our multimodal IAC‐PR model effectively integrated CT images with textual clinical information and significantly outperformed other models trained with individual or partial modalities in postoperative recurrence risk stratification. This model provides highly accurate recurrence risk assessment in early‐stage LUAD, enabling clinicians to tailor and adjust treatment strategies according to patients’ assigned risk levels.

Previous studies have shown that grade 3 LUADs harbor a higher prevalence of ALK rearrangements and KRAS mutations, greater tumor mutational burdens, and more frequent copy number amplifications compared with grades 1–2 [34, 35]. Recent work further highlighted the heterogeneity within grade 3 LUAD, identifying three distinct molecular subtypes based on transcriptomic and methylation data, each with different prognostic implications [36]. These findings suggest that integrative multi‐omics analysis could complement our system by uncovering clinicopathological and molecular correlates of recurrence risk, warranting further exploration. Moreover, incorporating histopathology whole‐slide images (WSIs) represents another promising direction. Transformers have demonstrated strong performance in analyzing WSIs, enabling extraction of diverse histological features for cancer diagnosis and prognosis [37]. Wang et al. developed an ensemble transformer‐based encoder to predict pathological subtypes directly from H&E‐stained WSIs in endometrial and colorectal cancers [38]. Extending our multimodal framework to include WSI data could further enhance recurrence risk stratification for early‐stage NSCLC patients.

There were several limitations to our study. First, the accurate prediction of MIA is highly valuable for guiding the timing of surgery; therefore, achieving a ternary classification of invasiveness remains a key objective for future research. Second, the proposed postoperative recurrence risk stratification system for T1N0M0 LUAD patients lacks explanatory insight at the molecular level. Third, incorporating additional information from other clinical indicators, such as WSI, may further enhance the accuracy of the IAC‐PR model. Fourth, longer term follow‐up data on postoperative recurrence will be essential to refine the risk stratification system, and we are actively continuing this work.

In conclusion, we developed a T1LUT system for T1N0M0 LUAD that operates at two critical junctures: preoperative identification of invasiveness and postoperative stratification of recurrence risk. The use of large‐scale, multicenter datasets supports the robustness and generalizability of our models. By combining multi‐task evaluation with multimodal data integration, this system provides a reliable, noninvasive tool with significant potential to improve clinical decision‐making and patient outcomes. Its future open‐source application across broader populations could further facilitate precision management of early‐stage lung cancer.

## 4 Materials and Methods

### 4.1 Patients and Data Collection

In this study, we collected medical records—including demographic characteristics, chest CT data, surgery‐related information, and pathologic findings—from six hospitals located in three provinces of China. Specifically, 2358 patients from Wuhan Union Hospital (Center A) between July 2019 and May 2023, 1502 patients from Shiyan Taihe Hospital (Center B) between March 2020 and December 2024, and 1171 patients from the Xiangyang Central Hospital (Center C) between May 2021 and December 2024 were enrolled in invasiveness classification as the training set. In addition, 884 patients from Renmin Hospital of Wuhan University (Center D) between January 2021 and December 2022, 358 patients from Renmin Hospital of Henan Province (Center E) between January 2024 and January 2025, and 205 patients from the First Affiliated Hospital of Guangzhou Medical University (Center F) between January 2024 and July 2024 were enrolled as three independent testing sets. The inclusion criteria for invasiveness identification were as follows: (1) a clinical diagnosis of stage T1N0M0 LUAD; (2) availability of preoperative chest CT images and reports within 3 months before surgery; and (3) a CT‐measured nodule diameter ≥ 4 mm and ≤ 3 cm. The exclusion criteria were as follows: (1) nodule diameter > 3 cm; (2) incomplete essential clinical data; (3) history of other malignant tumors; and (4) age <18 or >80 years.

For postoperative recurrence risk stratification, patients with stage T1N0M0 LUAD who were pathologically diagnosed with IAC and had at least 3 years of follow‐up after surgery were included. The last follow‐up date was May 31, 2025. A total of 1401 patients were enrolled from four hospitals: 595 from Center A, 380 from Center B, and 164 from Center C as the training set, and 262 from Center D as the independent testing set. Patient selection details are presented in Figure S6. This study was approved by the ethics committees of all participating hospitals: Wuhan Union Hospital (2022‐0649‐01), Shiyan Taihe Hospital (2022KS027), Xiangyang Central Hospital (2024‐057), Renmin Hospital of Wuhan University (WDRY2023‐K004), Henan Provincial People's Hospital (2023‐19), and the First Affiliated Hospital of Guangzhou Medical University (ES‐2025‐005‐01), and this study was conducted in accordance with the Declaration of Helsinki and waived the need for informed consent due to its observational design.

### 4.2 The Classification Criterion for Preoperative Invasiveness Identification and Postoperative Recurrence Risk Stratification

For invasiveness identification, each case was classified into one of the four invasive statuses: AAH, AIS, MIA, and IAC [1]. For analysis, AAH, AIS, and MIA were grouped together as the preinvasive/minimally invasive group, while IAC was defined as the invasive group [39]. For postoperative recurrence risk stratification, patients with IAC were further stratified into three recurrence‐risk categories—low, medium, and high—based on a combination of the IASLC grading system and follow‐up information. The IASLC grading system defines histologic grades as follows: Grade 1 (well differentiated), lepidic predominant tumors with < 20% high‐grade patterns (solid, micropapillary, or complex gland); Grade 2 (moderately differentiated), acinar or papillary predominant tumors, both with < 20% high‐grade patterns; and Grade 3 (poorly differentiated), any tumor containing ≥ 20% high‐grade patterns [15]. For stage T1N0M0 LUAD patients pathologically diagnosed with IAC, those who developed recurrence within 3 years after surgery were assigned to the high‐risk group, those without recurrence within 3 years after surgery but classified as grade 3 according to the IASLC grading system were assigned to the medium‐risk group, and the remaining patients—those without recurrence within 3 years after surgery and classified as grade 1 or 2—were assigned to the low‐risk group.

### 4.3 T1LUT‐1: Development of ViT‐Based Model to Identify the Invasiveness of Stage T1N0M0 LUAD

*Preprocessing of CT image data*: CT images collected from various hospitals were standardized to send to the models. Two radiologists (L.Z. and L.Y. with 15 and 20 years of clinical experience in lung CT interpretation, respectively) identified the axial slice showing the maximal diameter of the target nodule. Additionally, the long and short diameters of the targeted nodule on this 2D image layer were measured, and the coordinate of the central point of the targeted nodule was recorded. The DICOM format of CT images was converted to NIfTI format.

As detailed in the Supporting Information Methods, our initial development of a basic 2D ViT model (using 2D CT images) was an early attempt at invasiveness identification for T1N0M0 LUAD and the development of a 3D ResNet model, which was a traditional CNN‐based method served as a comparative analysis against ViT‐based method. Therefore, the model architecture was expanded into a 3D ViT to capture volumetric contextual information inherent to CT scans.

For 3D ViT model, a 3D cubic patch of 42 × 42 × 18 pixels as the region of interest was extracted with the central point of the targeted nodule as the central point of this patch. Then, the voxel spacing of this patch was standardized to 1 × 1 × 1 mm^3^ by trilinear interpolation. Data augmentation techniques, including rotation, scaling, flipping, and intensity transformation, were applied to the patches to enhance the generalization ability of the model [40].

*Development of an upgraded 3D ViT model*: The extracted patches of 42 × 42 × 18 pixels were input to the 3D patch embedding layer and cropped to smaller patches of 6 × 6 × 6 pixels. Each input image was divided into a number of 7 × 7 × 3 patches. Those patches were linearly transformed into 768‐dimensional embedding space. Before inputting to the transformer encoder layers, multiple randomly initialized learnable tokens have been added to enhance the expression ability of pulmonary nodule features.

To enhance the model's ability to discern fine‐grained medical features, drawing inspiration from methods such as Visual Prompt Tuning [41], Prompt‐CAM [42], and INterpretable TRansformer [43], M additional learnable parameters $P=\{p_{1},\text{…},p_{M}\}$ were introduced into the input patch token sequence, forming an extended sequence:

(1) $$X^{\prime }=\left[X;P\right]$$

 where X represents the patch token sequence of the original image and P denotes the set of learnable parameters. After processing by the encoder, the output corresponding to P was extracted as $X^{\prime }$ for downstream classification tasks. During training, an orthogonality loss $\mathrm{los}s_{\mathrm{Ortho}}$ was applied to constrain P, encouraging it to learn diverse and discriminative features. The $\mathrm{los}s_{\mathrm{Ortho}}$ was defined as:

(2) $$\;\mathrm{Los}s_{\mathrm{ortho}}=\frac{1}{M\left(M-1\right)}\;\sum_{i\neq j}\frac{\langle p_{i},p_{j}\rangle }{\;\left|\left|p_{i}\right|\right|\;\left|\left|p_{j}\right|\right|}$$

This design required no modifications to the original ViT architecture, offering strong modular flexibility.

The resulting sequence was processed by several transformer encoder layers consisting of multi‐head self‐attention and feed‐forward network modules for feature extraction and interaction [44]. The output CLS token, as the representation of global feature, was used to perform the final classification and input to a classification head, which was a multi‐layer perceptron consisting of fully connected layers and yielded a probability distribution across two classes: “AAH/AIS/MIA” and “IAC” for the pulmonary nodules via a Softmax activation function (Figure 3A).

**FIGURE 3:**
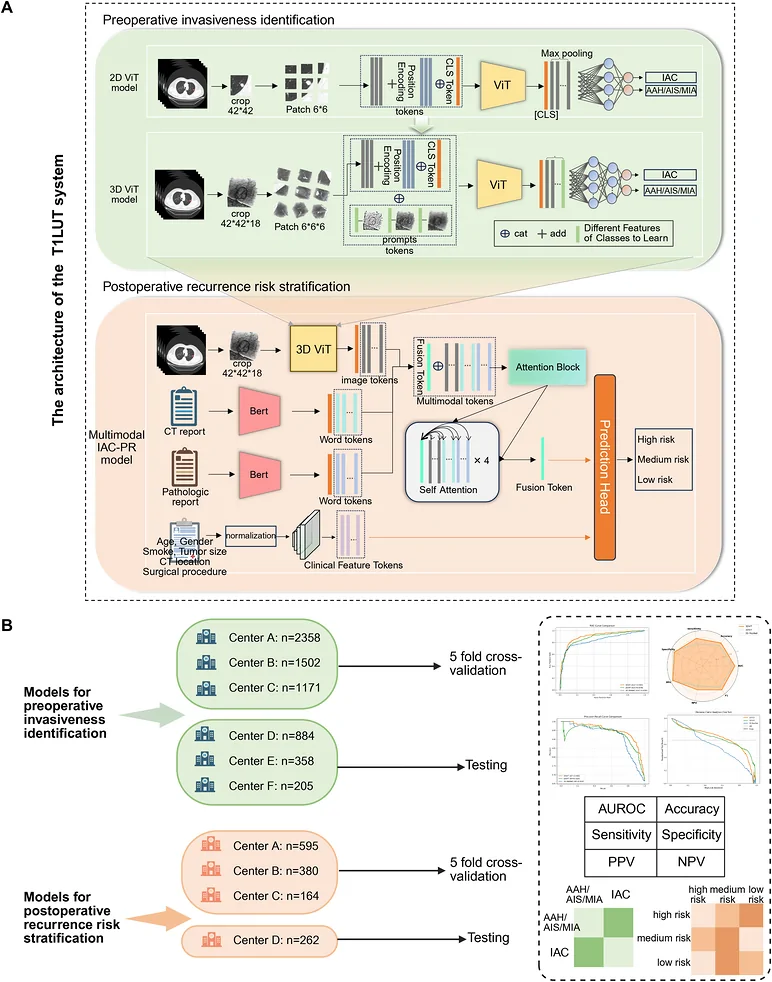
Study design for the cross‐validation and testing of the models in the T1LUT system. (A) 2D ViT model and 3D ViT model were constructed based on CT images by using ViT‐based architecture to predict the invasiveness of T1N0M0 LUAC patients preoperatively; CT images, CT reports, pathologic reports, and demographic and clinical information were integrated to develop the multi‐modal IAC‐PR model to stratify the postoperative recurrence risk for IAC patients, including high risk, medium risk and low risk. (B) Dataset consisting of Center A, Center B, and Center C was used to conduct fivefold cross‐validation, while three independent testing sets (Center D, Center E, and Center F) were used to test the performance of the models for preoperative invasiveness identification. And dataset collected from Center A, Center B, and Center C was used to conduct fivefold cross‐validation, while the Center D testing set was used to test the performance of the models for postoperative recurrence risk stratification. Abbreviations: IAC, invasive adenocarcinoma; LUAC, lung adenocarcinoma.

The detailed architecture of the ViT with extra learnable tokens in the 3D ViT model is shown in Figure S7. These tokens were designed to capture complementary discriminative cues. An orthogonality constraint was applied to reduce redundancy among tokens and promote feature decoupling, thereby widening the feature space margin between different invasiveness types.

In this stage, the model was optimized using a combined loss function consisting of cross‐entropy loss (*Loss_CE_*) and similarity loss (*Loss_ortho_*):

(3)

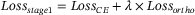




 where λ = 0.5. The AdamW optimizer was employed with a weight decay of 0.05. The initial learning rate was set to 5 × 10^−^^5^, and adjusted using a cosine annealing scheduler. A warm‐up was applied over 3 epochs to enhance training stability and generalization performance [45]. All hyperparameters were determined based on the training set performance.

### 4.4 T1LUT‐2: Development of Multimodal IAC‐PR Model Based on Transformer by Integrating Multimodal Clinical Data to Predict Postoperative Recurrence Risk of the Stage T1N0M0 LUAD Patients Pathologically Diagnosed With IAC

*Preprocessing of multimodal data*: The multimodal dataset comprised preoperative CT images, corresponding CT and pathology reports, and structured demographic and clinical variables (e.g., sex, age, smoking history, nodule location, and pathologic histologic pattern). The preprocessing of CT images was the same as the 3D ViT model in invasiveness identification. For textual data, we encoded CT reports and pathologic reports using a BERT encoder that was pre‐trained on Chinese medical corpora and fine‐tuned on our specific task [46]. To process the pathologic histologic patterns (e.g., papillary, lepidic, and acinar) mentioned in the pathology reports, we employed a semi‐automatic structured extraction pipeline powered by DeepSeek‐V3 [47]. This approach used standardized prompts for few/zero‐shot information extraction, outputting candidate histologic patterns along with corresponding evidence segments. The results were finally reviewed and corrected by an experienced pathologist (NZ).

*Encoder architecture of the multimodal IAC‐PR model*: Three independent encoders constituted the architecture of the multimodal IAC‐PR model, including image encoder, textual encoder, and clinical and demographic feature encoder. *Image encoder*: The well‐trained 3D ViT model in invasiveness identification was used to extract features from CT volume data and output a global volume‐level representation. Its parameters were initialized with weights from the former task of invasiveness identification and frozen during this task. *Textual encoder*: A BERT model pre‐trained on Chinese medical corpora encoded both pathologic reports and CT reports. During training, only the top two Transformer blocks were unfrozen and fine‐tuned, while the remaining parameters remained frozen. *Clinical and demographic feature encoder*: Structured clinical and demographic variables, including age, gender, smoking history, tumor size, CT location, and surgical procedure, were standardized and then input to a multi‐layer perceptron to obtain embedded representations. To achieve cross‐modal alignment, the representations from each of the three encoders were mapped into a unified dimensional space via separate linear projection heads.

*Multimodal integration of the multimodal IAC‐PR model*: The second part of the T1LUT system was to stratify postoperative recurrence risk in stage T1N0M0 LUAD patients pathologically diagnosed with IAC. Owing to the relatively low recurrence rate in this population, the distribution of positive and negative cases was imbalanced. Furthermore, image‐only models demonstrated notable performance degradation and poor generalization across independent testing sets, indicating insufficient robustness for recurrence prediction. Therefore, a multimodal integration strategy—aligning and fusing complementary information from CT images, textual reports, and clinical data—was adopted to enhance model performance (Figure 3A).

In our multimodal fusion framework, features from different modalities were first encoded by their dedicated encoders. The resulting feature vectors were then concatenated, and a fusion token was appended to this combined sequence. The entire fused sequence—comprising all modality features and the fusion token—was input to a lightweight transformer module. Within this module, the self‐attention mechanism captured high‐order interactions among modalities. During this process, the fusion token engaged in bidirectional information exchange with all modality features, thereby capturing critical and complementary cross‐modal information. The updated fusion token from the transformer output was used as the global multimodal representation and was passed to a classification head to predict the patient's risk of postoperative recurrence (Figure 3A).

Effective multimodal fusion is critical for optimizing downstream task performance. Building on the strategy of incorporating additional learnable tokens for processing CT images in invasiveness identification (Figure S7), we extended this mechanism to the multimodal scenario. Specifically, by adding learnable tokens to the patch token sequence, the model can capture diverse and discriminative features through self‐attention mechanisms enhanced by similarity constraints. Likewise, introducing trainable fusion tokens into the multimodal feature sequence enabled the model to extract and aggregate informative representations across modalities via self‐attention mechanisms.

### 4.5 Evaluation Metrics About Model Performance

For the evaluation of models’ performance on invasiveness prediction and risk stratification of postoperative recurrence, the area under receiver operating characteristic curve (AUROC), the area under precision‐recall curve (AUPRC), accuracy, sensitivity, specificity, positive predictive value (PPV), negative predictive value (NPV), F1 score, and confusion matrices were employed. Moreover, decision curve analysis (DCA) was performed to estimate the clinical utility of the models by evaluating their net benefits when applied to a test set at different threshold probabilities (Figure 3B).

### 4.6 Quantification and Statistical Analysis

During the process of developing models in the T1LUT system, a fivefold cross‐validation method was employed to ensure that the generalization of the models was fully evaluated. For the postoperative recurrence risk stratification, an intra‐batch oversampling strategy was employed within each training batch to ensure that the ratio of positive and negative samples is at least 3:7, thereby enhancing the models’ sensitivity to minority classes. Continuous variables were expressed as the median (interquartile range [IQR]), whereas categorical variables were expressed using relative frequencies and proportions. As appropriate, the *t*‐test or the Mann–Whitney *U*‐test was performed for continuous variables, and the chi‐square test or Fisher's exact test for categorical variables. All tests were two‐sided, and *p* < 0.05 indicated statistical significance. All experiments in this study were conducted on a CentOS Linux‐based server equipped with two NVIDIA A100 80 GB GPUs (total GPU memory: 160 GB), using Python 3.8 and PyTorch 2.1.1 with CUDA 11.8. The details about the parameter settings of the models in the T1LUT system are shown in the Supporting Information Methods.

## Author Contributions

**Xueyun Tan**: investigation, data curation, methodology, writing – original draft. **Hui Xia**: data curation, methodology, writing – original draft. **Delong Li**: methodology, data curation, writing – original draft. **Meifang Wang**: data curation, investigation. **Na Zhan**: conceptualization, methodology, data curation. **Yan Huang**: methodology, data curation. **Mengyuan Liang**: data curation, investigation, methodology. **Pei Ma**: data curation, investigation. **Xiao Wang**: data curation, investigation. **Yaqi Cao**: data curation, investigation. **Linpeng Shi**: methodology, supervision. **Liang Zhang**: data curation. **Xiaoling Liu**: methodology. **Lian Yang**: data curation. **Quan Kong**: methodology, supervision, writing – review and editing. **Sufei Wang**: conceptualization, project administration, writing – review and editing. **Yang Jin**: conceptualization, funding acquisition, supervision, writing – review and editing.

## Funding

This work was supported by the National Science and Technology Major Project of the Ministry of Science and Technology of China (Nos. 2023ZD0509900 and 2023ZD0509905), National Natural Science Foundation of China (Nos. 82400121, 82570136, and 82330003), Major Project of the Department of Science and Technology of Hubei (Nos. 2025DJA003 and 2022BCA016), and Natural Science Foundation of Hubei Province (No. 2025AFD181).

## Ethics Statement

This study was conducted under the approval of Wuhan Union Hospital (2022‐0649‐01), Shiyan Taihe Hospital (2022KS027), Xiangyang Central Hospital (2024‐057), Renmin Hospital of Wuhan University (WDRY2023‐K004), Henan Provincial People's Hospital (2023‐19), and the First Affiliated Hospital of Guangzhou Medical University (ES‐2025‐005‐01). And this study was conducted in accordance with the Declaration of Helsinki and waived the need for informed consent due to its observational design.

## Conflicts of Interest

Xiaoling Liu is an employee of Neusoft Ltd. He does not hold equity interests, including stocks or stock options, in Neusoft Ltd. He is not named as an inventor on patents or patent applications related to the work reported in this article. He reports no stocks, stock options, patents, patent applications, consulting fees, or other financial interests related to the work. All other authors declare no conflicts of interest.

## Supporting information




**Supporting File 1**: mco271055‐sup‐0001‐SuppMat.pdf.

## Data Availability

The data that support the findings of this study are not openly available due to reasons of sensitivity and are available from the corresponding author upon reasonable request. The code for the models in the T1LUT system is deposited in GitHub at https://github.com/Robotld/3DVM, which is publicly accessible.
