# Supplementary material for: Transformer‐Based Preoperative Invasiveness Identification and Postoperative Recurrence Risk Stratification in Stage T1N0M0 Lung Adenocarcinoma: A Multi‐Center Cohort Study
Source: MedComm (2020). 2026 Oct 11;7(10):e71055. doi: 10.1002/mco2.71055 (PMC13655376; doi:10.1002/mco2.71055)
Supplement: Supplementary file 1 [file MCO2-7-e71055-s001.pdf]

Transformer-based preoperative invasiveness identification and postoperative recurrence risk stratification in stage T1N0M0 lung adenocarcinoma: a multi-center cohort study

Xueyun Tan, MD[1,4]*, Hui Xia, MD[1,2,3]*, Delong Li, MS[5]*, Meifang Wang, MS[6]*, Na Zhan, MS[7]*, Yan Huang, MS[8]*, Mengyuan Liang, PhD[9]*, Pei Ma, PhD[10]*, Xiao Wang, MS[6], Yaqi Cao, MS[1,2,3], Linpeng Shi, MD[1,2,3], Liang Zhang, MS[11], Xiaoling Liu, PhD[12]*, Lian Yang, PhD[13], Quan Kong, MS[14#], Sufei Wang, PhD[1,2,3#], Yang Jin, PhD[1,2,3#]

[1] Department of Respiratory and Critical Care Medicine, Hubei Province Clinical Research Center for Major Respiratory Disease, Union Hospital, Tongji Medical College, Huazhong University of Science and Technology, Wuhan, China

[2] Hubei Province Key Laboratory of Biological Targeted Therapy, MOE Key Laboratory of Biological Targeted Therapy, Union Hospital, Tongji Medical College, Huazhong University of Science and Technology, Wuhan, China

[3] Hubei Province Engineering Research Center for Tumor-Targeted Biochemotherapy, Union Hospital, Tongji Medical College, Huazhong University of Science and Technology, Wuhan, China

[4] Department of Respiratory and Critical Care Medicine, Zhongnan Hospital of Wuhan University, Wuhan, China

[5] Hubei Key Laboratory of Optical Information and Pattern Recognition, School of Optical Information and Energy Engineering, Wuhan Institute of Technology, Wuhan, China

[6] Department of Respiratory and Critical Care Medicine, Shiyan Key Laboratory of Major Chronic Respiratory Disease, Taihe Hospital, Hubei University of Medicine, Shiyan, China

[7] Department of Pathology, Renmin Hospital of Wuhan University, Wuhan, China

[8] Department of Respiratory and Critical Care Medicine, Xiangyang Central Hospital, Affiliated Hospital of Hubei University of Arts and Science, Xiangyang, China

[9] Department of Respiratory and Critical Care Medicine, Henan Provincial People's Hospital, Zhengzhou University, Zhengzhou, China

[10] Department of Respirology, Guangzhou Institute of Respiratory Health, The First Affiliated Hospital of Guangzhou Medical University, State Key Laboratory of Respiratory Diseases, Guangzhou, China

[11] Department of Radiology, Renmin Hospital of Wuhan University, Wuhan, China

[12] Institute of Research and Clinical Innovations, Neusoft Medical Systems Co. Ltd, Wuhan, China

[13] Department of Radiology, Union Hospital, Tongji Medical College, Huazhong University of Science and Technology, Wuhan, China

[14] Hubei Key Laboratory of Optical Information and Pattern Recognition, Wuhan Institute of Technology, Wuhan, China

*These authors contributed equally to this work

[#]Correspondence：

Yang Jin (whuhjy@126.com)

Department of Respiratory and Critical Care Medicine, Hubei Province Clinical Research Center for Major Respiratory Disease, NHC Key Laboratory of Pulmonary Diseases, Hubei Province Engineering Research Center for Tumor-Targeted Biochemotherapy, Hubei Province Key Laboratory of Biological Targeted Therapy, MOE Key Laboratory of Biological Targeted Therapy, Union Hospital, Tongji Medical College, Huazhong University of Science and Technology, Wuhan, Hubei, 430022, China

Sufei Wang (wangsufei0@163.com)
Department of Respiratory and Critical Care Medicine, Hubei Province Clinical Research Center for Major Respiratory Disease, NHC Key Laboratory of Pulmonary Diseases, Hubei Province Engineering Research Center for Tumor-Targeted Biochemotherapy, Hubei Province Key Laboratory of Biological Targeted Therapy, MOE Key Laboratory of Biological Targeted Therapy, Union Hospital, Tongji Medical College, Huazhong University of Science and Technology, Wuhan, Hubei, 430022, China
Quan Kong (witkongquan@wit.edu.cn)
Hubei Key Laboratory of Optical Information and Pattern Recognition, Wuhan Institute of Technology, Wuhan, Hubei, 430205, China

Table S1. Model performance metrics of the 3D ViT, the 3D ViT (only CLS), the 3D ResNet, and the 2D ViT models for invasiveness identification in three independent testing sets

Model	Index	Testing sets<br>Center D (n=884)	Center E (n=358)	Center F (n=205)
**3D ViT**	AUROC	0.896 (0.874-0.920)	0.906 (0.873-0.936)	0.884 (0.827-0.936)
	Accuracy	0.807 (0.779-0.831)	0.771 (0.725-0.811)	0.839 (0.783-0.883)
	Sensitivity	0.762 (0.726-0.795)	0.937 (0.891-0.965)	0.853 (0.756-0.916)
	Specificity	0.895 (0.855-0.925)	0.612 (0.540-0.680)	0.831 (0.757-0.886)
	PPV	0.935 (0.910-0.954)	0.698 (0.636-0.753)	0.744 (0.643-0.825)
	NPV	0.654 (0.607-0.699)	0.911 (0.847-0.949)	0.908 (0.842-0.948)
**3D ViT (only CLS)**	AUROC	0.892 (0.869-0.914)	0.837 (0.796-0.878)	0.884 (0.821-0.939)
	Accuracy	0.752 (0.723-0.780)	0.737 (0.690-0.779)	0.820 (0.761-0.866)
	Sensitivity	0.665 (0.626-0.702)	0.781 (0.712-0.843)	0.853 (0.756-0.916)
	Specificity	0.926 (0.890-0.950)	0.696 (0.621-0.760)	0.800 (0.723-0.860)
	PPV	0.947 (0.921-0.965)	0.710 (0.644-0.774)	0.711 (0.610-0.795)
	NPV	0.582 (0.537-0.625)	0.770 (0.706-0.828)	0.904 (0.837-0.946)
**3D ResNet**	AUROC	0.836 (0.809-0.863)	0.835 (0.793-0.876)	0.861 (0.803-0.920)
	Accuracy	0.734 (0.704-0.762)	0.751 (0.704-0.793)	0.829 (0.772-0.875)
	Sensitivity	0.631 (0.591-0.669)	0.697 (0.625-0.760)	0.707 (0.596-0.798)
	Specificity	0.939 (0.906-0.961)	0.803 (0.740-0.854)	0.900 (0.836-0.941)
	PPV	0.954 (0.928-0.971)	0.772 (0.701-0.831)	0.803 (0.692-0.881)
	NPV	0.562 (0.518-0.605)	0.735 (0.670-0.791)	0.842 (0.772-0.893)
**2D ViT**	AUROC	0.879 (0.856-0.900)	0.837 (0.796-0.878)	0.881 (0.829-0.928)
	Accuracy	0.751 (0.724-0.779)	0.737 (0.690-0.779)	0.819 (0.766-0.873)
	Sensitivity	0.659 (0.621-0.696)	0.781 (0.712-0.843)	0.760 (0.667-0.861)
	Specificity	0.933 (0.903-0.960)	0.696 (0.621-0.760)	0.854 (0.793-0.913)
	PPV	0.951 (0.926-0.972)	0.710 (0.644-0.774)	0.750 (0.652-0.842)
	NPV	0.579 (0.537-0.623)	0.770 (0.706-0.828)	0.861 (0.800-0.918)

Data are presented as mean (95% CI).

AUROC, the area under receiver operating characteristic curve; PPV, positive predictive value; NPV, negative predictive value.

Table S2. Subgroup analysis of the 3D ViT model for invasiveness identification in testing sets based on tumor size on CT

	Testing sets (n=1447)		
	<10mm (n=433)	10-20mm (n=803)	20-30mm (n=211)
**IAC**	71 (16.3%)	559 (69.6%)	208 (98.6%)
**MIA**	216 (50.0%)	189 (23.5%)	3 (1.4%)
**AAH/AIS**	146 (33.7%)	55 (6.9%)	0 (0%)
**AUROC**	0.695 (0.623-0.767)	0.834 (0.806-0.862)	0.814 (0.483-1.000)
**Accuracy**	0.785 (0.744-0.821)	0.761 (0.730-0.789)	0.896 (0.847-0.930)
**Sensitivity**	0.408 (0.302-0.525)	0.758 (0.721-0.792)	0.899 (0.851-0.933)
**Specificity**	0.859 (0.819-0.891)	0.766 (0.709-0.815)	0.667 (0.208-0.939)
**PPV**	0.362 (0.266-0.472)	0.881 (0.850-0.907)	0.995 (0.970-0.999)
**NPV**	0.881 (0.843-0.911)	0.581 (0.526-0.633)	0.087 (0.024-0.268)

Data are presented as mean (95% CI).

IAC, invasive adenocarcinoma; MIA, minimally invasive adenocarcinoma; AAH, atypical adenomatous hyperplasia; AIS, adenocarcinoma in situ; AUROC, the area under receiver operating characteristic curve; PPV, positive predictive value; NPV, negative predictive value.

Table S3. Comparison of model performance metrics for postoperative stratification of recurrence risk in Center D testing set

Index	Multimodal IAC-PR	3D ViT (Image only model)	Image and demographic modal	Text and demographic modal
**AUROC**	0.807 (0.750-0.860)	0.637 (0.570-0.701)	0.641 (0.573-0.710)	0.726 (0.662-0.786)
**Accuracy**	0.805 (0.753-0.849)	0.435 (0.376-0.496)	0.408 (0.351-0.469)	0.767 (0.712-0.814)
**Sensitivity**	0.624 (0.551-0.693)	0.438 (0.356-0.521)	0.400 (0.320-0.486)	0.554 (0.482-0.625)
**Specificity**	0.848 (0.809-0.889)	0.747 (0.713-0.782)	0.737 (0.704-0.773)	0.821 (0.779-0.857)
**PPV**	0.613 (0.535-0.691)	0.411 (0.370-0.453)	0.395 (0.357-0.439)	0.537 (0.447-0.633)
**NPV**	0.844 (0.805-0.884)	0.708 (0.686-0.730)	0.699 (0.679-0.722)	0.817 (0.776-0.855)

Data are presented as mean (95% CI).

AUROC, the area under receiver operating characteristic curve; PPV, positive predictive value; NPV, negative predictive value.

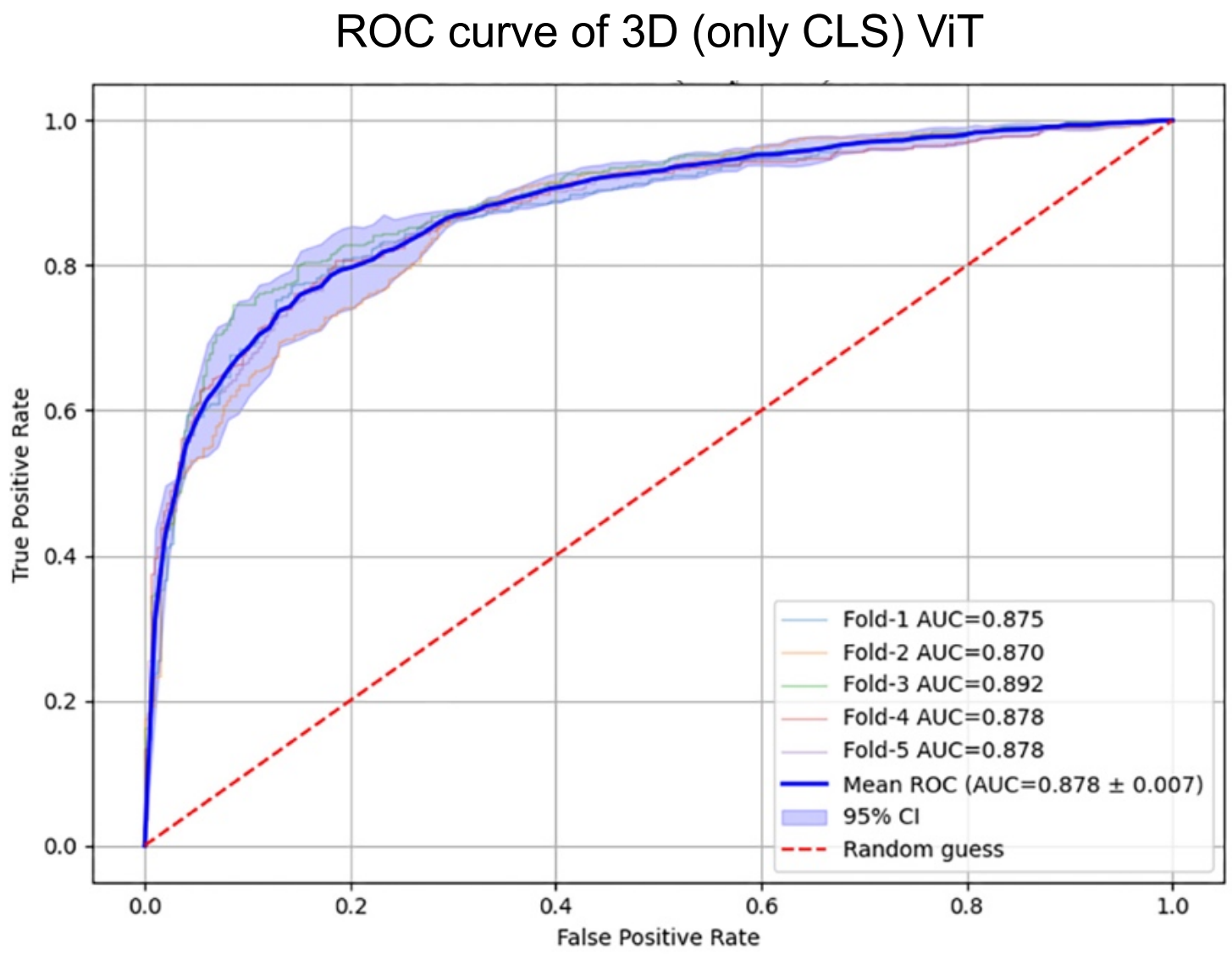

Figure S1. A 5-fold cross-validation was conducted during the training of the 3D (only CLS) ViT model for invasiveness identification, and the AUROCs were plotted along with the 95% CI region.

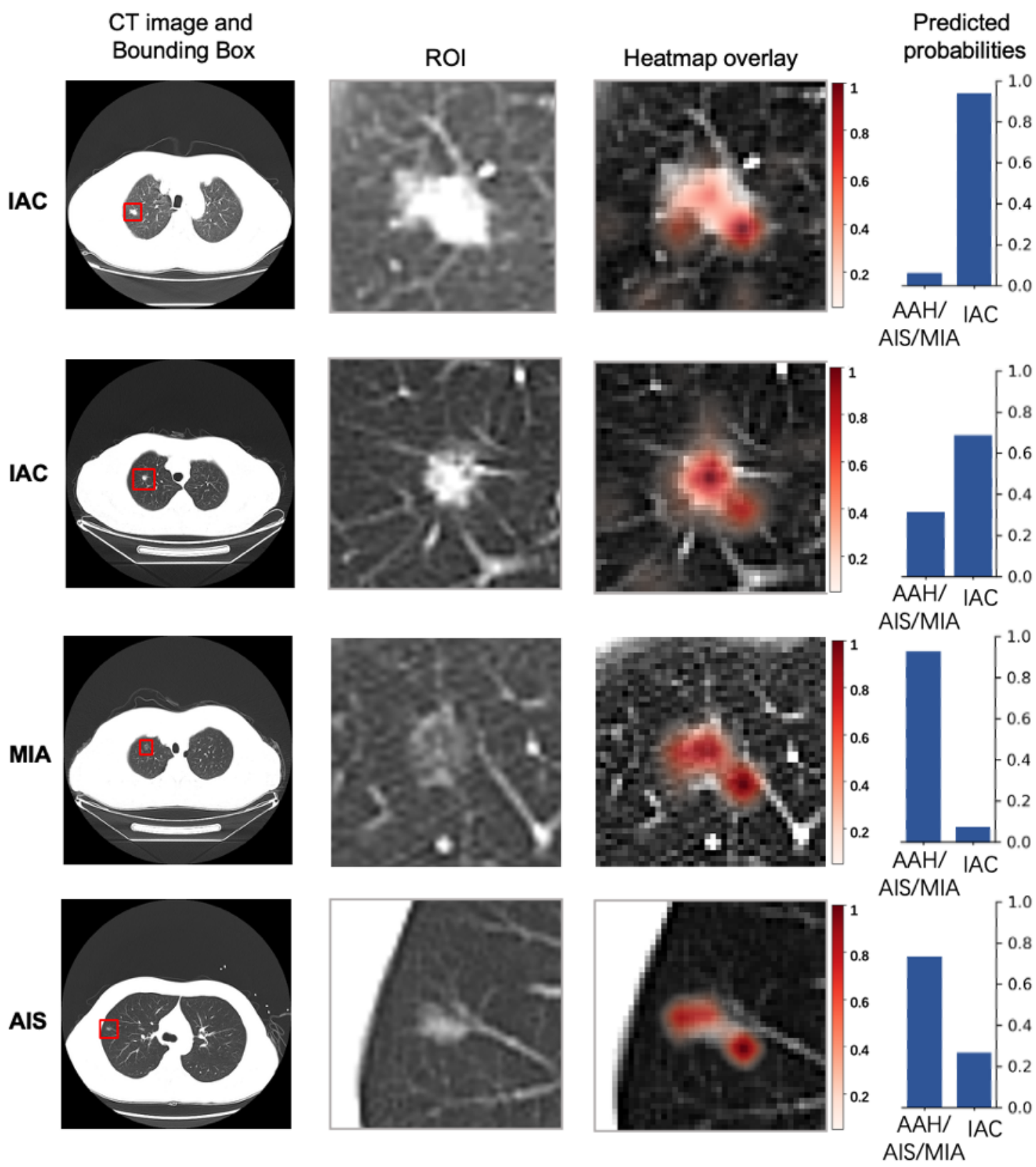

Figure S2. CT images and corresponding heatmaps along with the predicted invasiveness for four representative cases, where each row corresponds to a patient with IAC, or MIA, or AIS defined by pathologic diagnosis. Invasiveness types were correctly predicted for all four cases.

ROI, region of interest; IAC, invasive adenocarcinoma; MIA, minimally invasive adenocarcinoma; AIS, adenocarcinoma in situ.

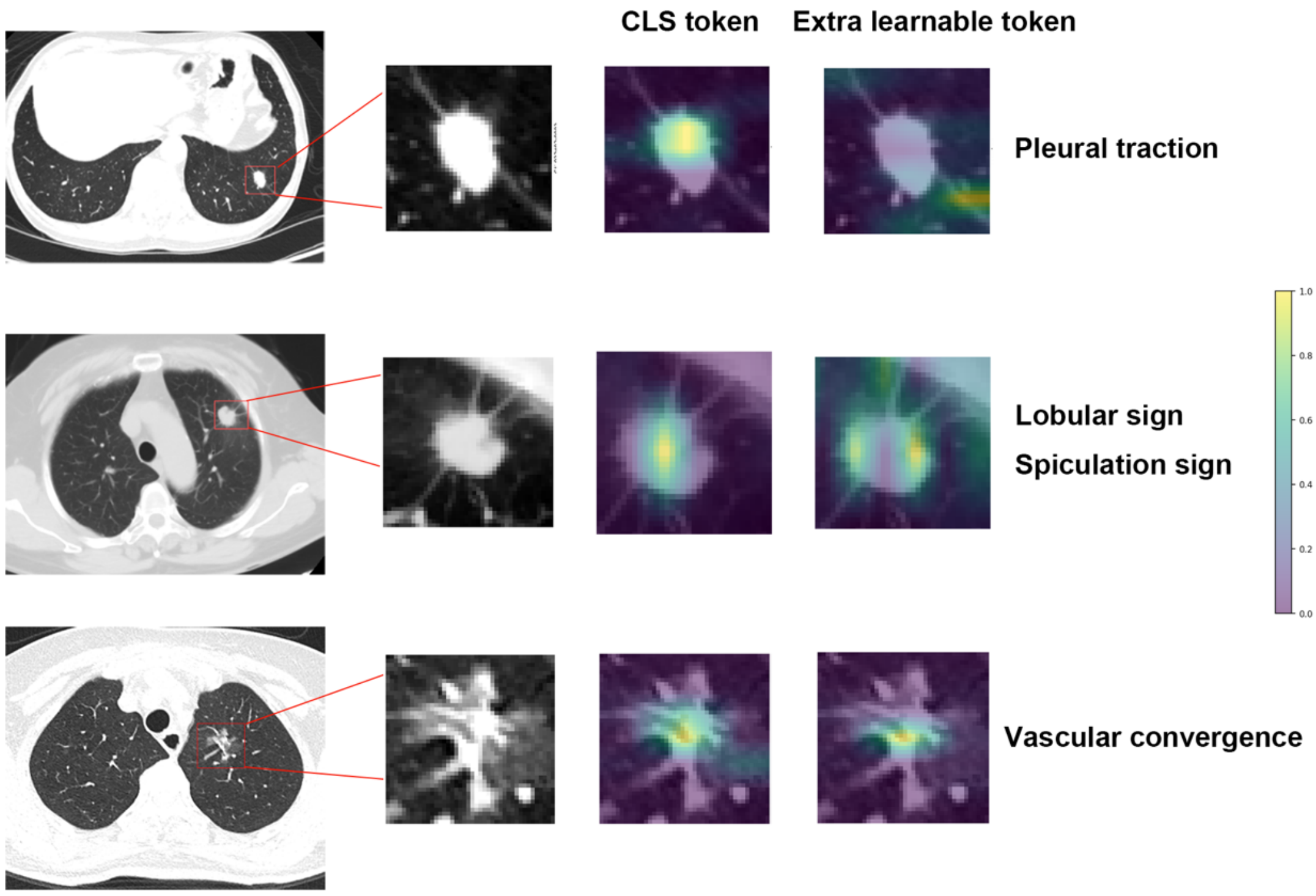

Figure S3. CT images with representative radiological features (pleural retraction, lobular sign, spiculation, and vascular convergence), along with corresponding heatmaps and a comparison of attention regions between the CLS token and extra learnable token.

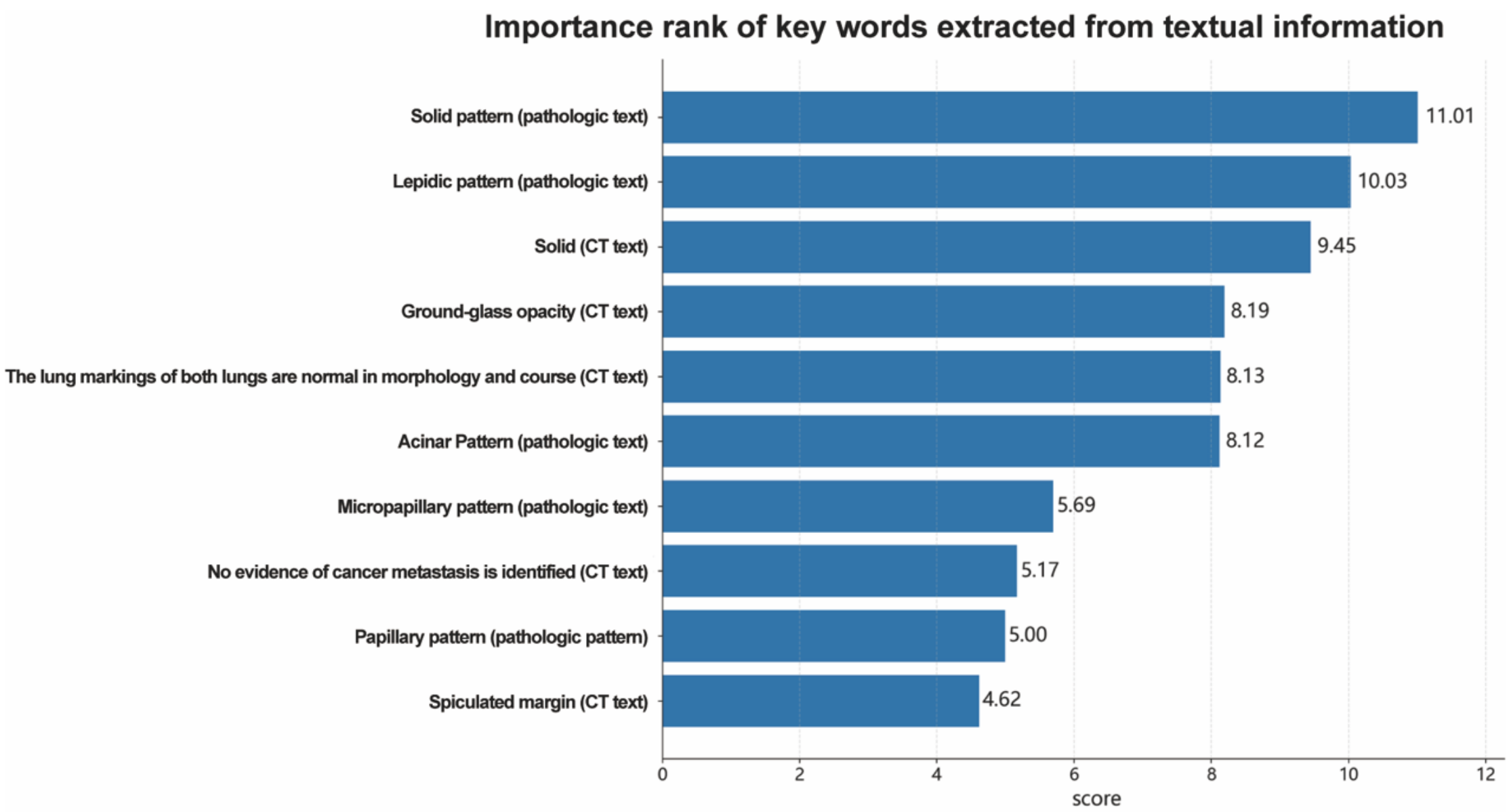

Figure S4. Importance rank for the IAC-PR model performance of keywords extracted from textual information including CT reports and pathologic reports, and Chinese were translated to English in the plot.

A

	Training set (n=738)	Testing set (n=124)
**High-risk**	57 (7.7%)	13 (10.5%)
**Medium-risk**	0	0
**Low-risk**	681 (92.3%)	111 (89.5%)
**AUROC**	0.842 (0.790-0.887)	0.714 (0.509-0.873)
**Accuracy**	0.780 (0.749-0.809)	0.839 (0.774-0.903)
**Sensitivity**	0.724 (0.607-0.839)	0.538 (0.267-0.833)
**Specificity**	0.785 (0.755-0.814)	0.874 (0.811-0.938)
**PPV**	0.223 (0.164-0.282)	0.333 (0.125-0.565)
**NPV**	0.971 (0.956-0.984)	0.942 (0.897-0.981)

B

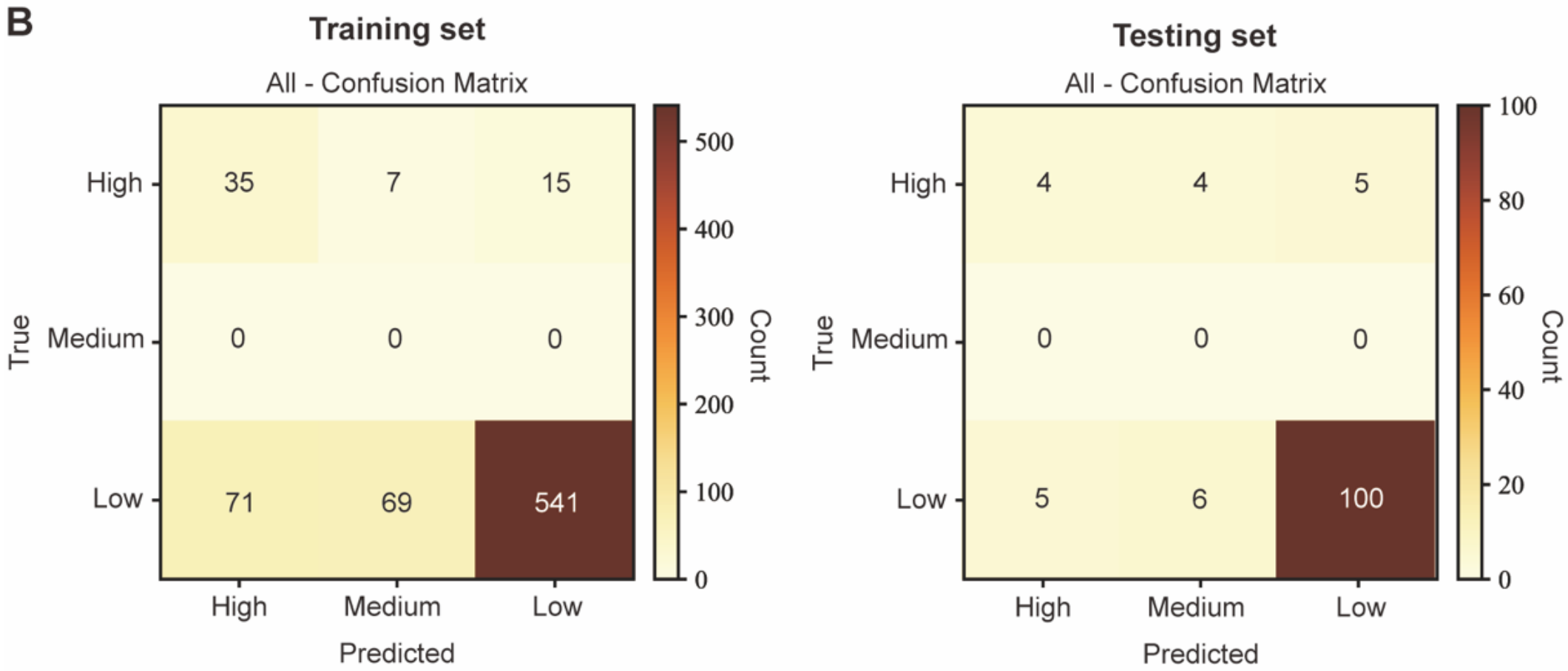

Figure S5. Subgroup analysis of the multimodal IAC-PR model for postoperative recurrence risk stratification in training and testing sets for Grade 2 patients.
(A) Predictive performance indexes in training and testing sets.
(B) Confusion matrices for postoperative recurrence risk stratification in training and testing set.

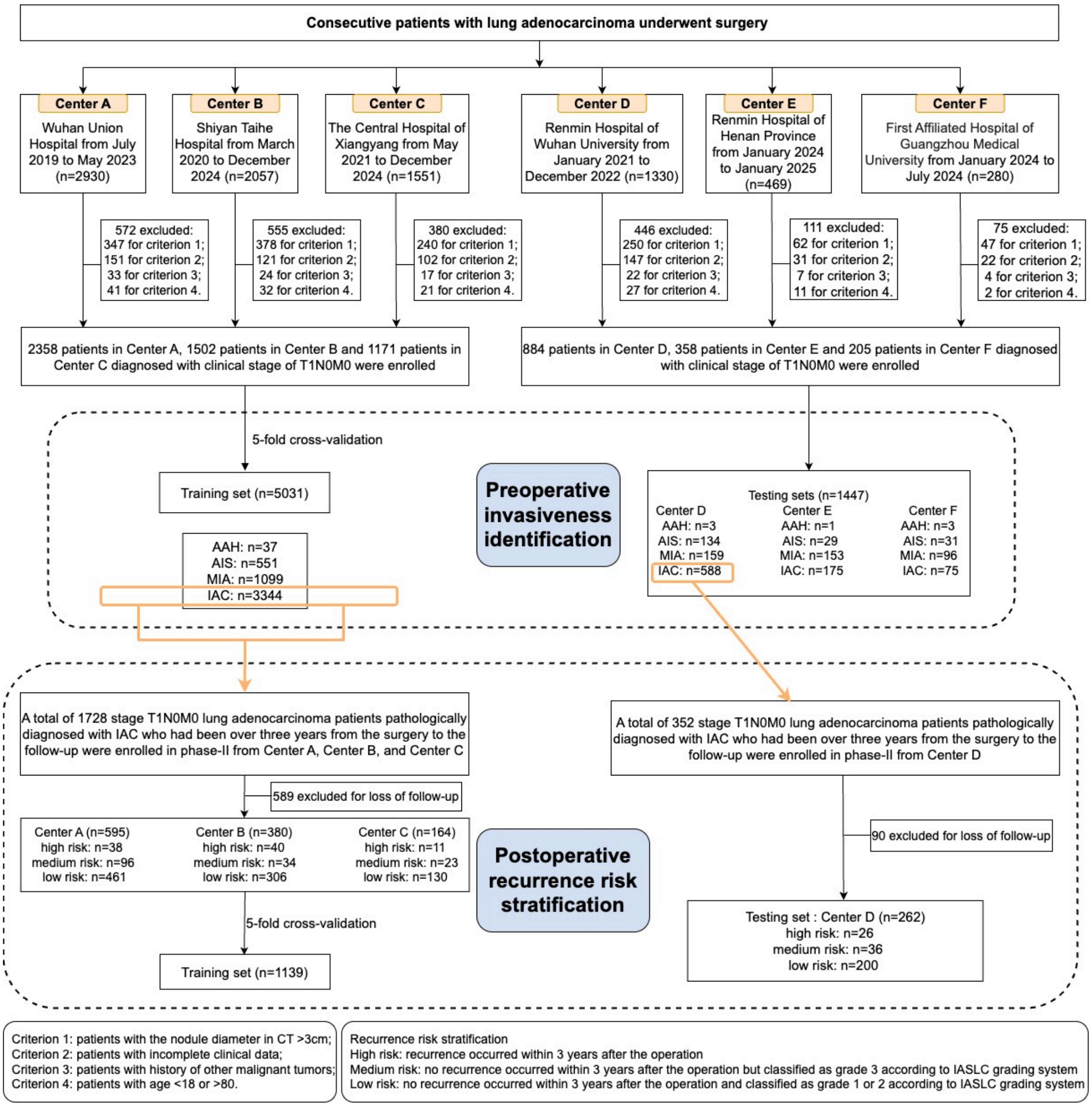

Figure S6. Flowchart of patient selection

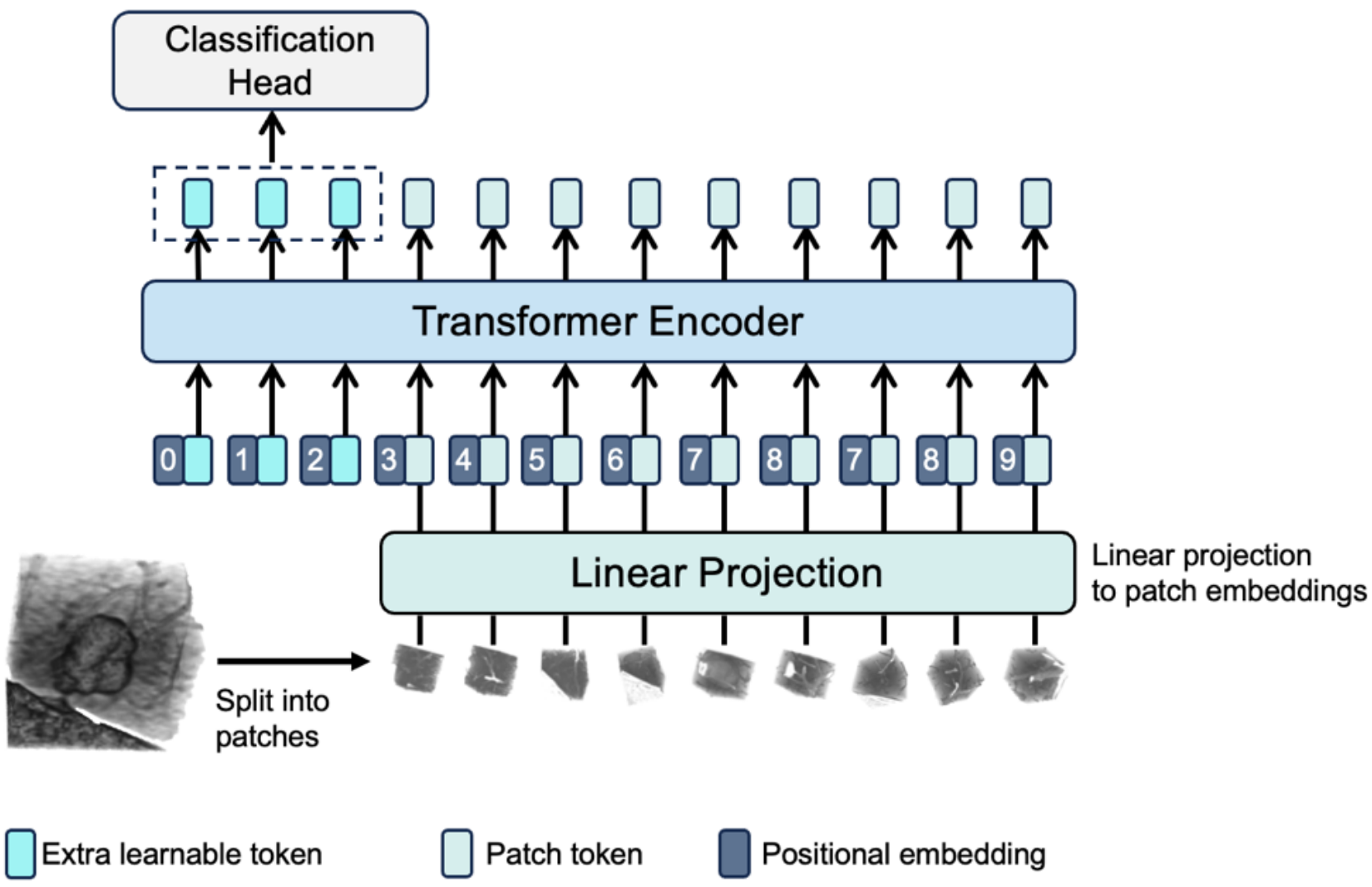

Figure S7. The detailed architecture of ViT in the 3D ViT model. Among the extra learnable tokens, the first is used to learn global features, and the remaining ones are used to learn local features.

Supplementary Methods

Development of basic 2D ViT model to identify the invasiveness of stage T1N0M0 LUAD

For 2D ViT model, a square patch of 42×42 pixels is extracted with the central point of the targeted nodule as the central point of this patch. This patch as the region of interest includes the nodule body and its local context information. The data augmentation techniques are the same as the 3D ViT model. The extracted patches of 42×42 pixels are input to the 2D patch embedding layer and cropped to smaller patches of 6×6 pixels. Each input image was divided into a number of 7×7 patches. Those patches are linearly transformed into 768-dimensional embedding space. And a learned class token, denoted as [CLS], is prepended to the sequence to aggregate global semantic information. Then, learnable positional embeddings are added to the patch embeddings to retain spatial relationships. The resulting sequence is processed by several transformer encoder layers consisting of multi-head self-attention and feed-forward network modules for feature extraction and interaction[1]. The output CLS token as the representation of global feature is used for performing the final classification and input to a classification head, which is a multi-layer perceptron consisting of fully-connected layers and yields a probability distribution across two classes: "AAH/AIS/MIA" and "IAC" for the pulmonary nodules via a Softmax activation function.

Note that before the training stage, both 2D ViT model and 3D ViT model are initialized using some parameters of the RAD-DINO model. RAD-DINO is a vision transformer model trained to encode chest X-rays using the self-supervised learning method DINOv2[2]. Since the RAD-DINO model is pre-trained on large-scale chest X-ray image datasets, its CLS token may carry bias information associated with the source domain. Zou et al.[3] indicated that randomly initializing the CLS token in ViT architectures can effectively mitigate interference from source domain features during target domain adaptation and improve downstream task performance. Inspired by this finding, we randomly initialize the CLS token when loading the pre-trained weights to enhance the model's generalization capability in the target domain.

The 3D ResNet model derived from C-Lung-RADS as the comparative model

For invasiveness identification, 3D ResNet101 was used as one of the baseline models for comparative validation. To ensure a fair comparison, the data preprocessing, training/validation/testing strategies, and hyperparameter configurations followed the recommended settings of C-Lung-RADS[4]. All models were trained and evaluated under the same training/validation/test splits, and identical data augmentation techniques, loss functions, and evaluation scripts were applied.

The parameter setting of models for the T1LUT system

**Preoperative invasiveness identification**	**Postoperative recurrence risk stratification**
num_classes: 2	num_classes: 3
model:<br>params:<br>image_size: [96, 96, 96]<br>patch_size: 6<br>dim: 768<br>depth: 6<br>heads: 6 # 6 8 12<br>mlp_dim: 128<br>mlp_num: 1 # 1 2 3<br>pool: mean<br>cpt_num: 0 # 2 5 10 50 100	model:<br>params:<br>image_size: [96, 96, 96]<br>patch_size: 6<br>dim: 768<br>depth: 6<br>heads: 6<br>mlp_dim: 128<br>mlp_num: 2<br>pool: 'max'<br>cpt_num: 5<br>bert_model_name:<br>'E:\workplace\3D\pred_model\bert_chinese'<br>tokenizer_path:<br>'E:\workplace\3D\pred_model\bert_chinese'<br>text_feature_dim: 768<br>demographic_dim: 10<br>fusion_dim: 384<br>fusion_transformer_heads: 4<br>fusion_transformer_layers: 4<br>dropout: 0.5<br>prompt_num: 0<br>fusion_method: "concat" # attention, concat, sum
training:<br>device: cuda<br>random_seed: 42<br>batch_size: 32<br>num_epochs: 20<br>num_workers: 4<br>use_amp: true<br>cache_dataset: true	training:<br>device: "cuda"<br>random_seed: 42<br>batch_size: 32<br>num_epochs: 20<br>num_workers: 2<br>use_amp: true<br>cache_dataset: True

warmup_epochs: 3<br>warmup_type: linear<br>max_grad_norm: 1.0<br>crop_size: [42,42,18] # [H, W, D]<br>augment: true<br>pretrained_path:<br>"E:\\workplace\\3D\\pred_model\\rad_dino"<br>frozen: false<br>frozen1: false<br>frozen2: false	warmup_epochs: 3<br>warmup_type: "linear"<br>max_grad_norm: 1.0<br>crop_size: [42, 42, 18]<br>augment: true<br>pretrained_path:<br>'E:\workplace\3D\src\mul_train_output\ 全 数据训练_多模态用\best_auc_model_0.pth'<br>bert_pretrained_path:<br>'E:\workplace\3D\pred_model\bert_chinese'<br>frozen1: true<br>frozen2: true<br>frozen_prompt: false
losses:<br>CrossEntropyLoss:<br>enabled: True<br>weight: 1.0<br>params:<br>weight: [1.0, 1.0]<br>reduction:<br>label_smoothing:<br>FocalLoss:<br>enabled: false<br>weight: 1.0<br>params:<br>gamma: 2.0<br>alpha: 0.25<br>BoundaryFlowLoss:<br>enabled: false<br>weight: 1.0<br>params:<br>weight: 0.7<br>margin: 0.3<br>SimilarityLoss:<br>enabled: false<br>params:<br>low: 0.3<br>high: 0.7	losses:<br>CrossEntropyLoss:<br>enabled: true<br>weight: 1.0<br>params: [5]<br>FocalLoss:<br>enabled: false<br>weight: 1.0<br>params:<br>gamma: 2.0<br>alpha: 0.25<br>BoundaryFlowLoss:<br>enabled: true<br>weight: 1.0<br>params:<br>weight: 0.5<br>margin: 0.3<br>SimilarityLoss:<br>enabled: false<br>params:<br>low: 0.3<br>high: 0.7<br>MultitaskLoss:<br>enabled: true<br>recurrence_weight: 1.0<br>axis_weight: 0.3
cross_validation:<br>n_splits: 5<br>shuffle: true<br>optimizer:<br>name: AdamW	cross_validation:<br>n_splits: 5<br>shuffle: true<br>negative_ratio: 12<br>pos_ratio: 0.3

params:<br>lr: 0.000005<br>weight_decay: 0.001<br>scheduler:<br>name: CosineAnnealingLR<br>params:<br>T_max: 20<br>eta_min: 1e-6	minority_multiplier: 10<br>use_clustering: false<br>clustering_method: "kmeans"<br>feature_extraction_batch_size: 32<br>optimizer:<br>name: "AdamW"<br>params:<br>lr: 0.00001<br>weight_decay: 0.01<br>scheduler:<br>name: "CosineAnnealingLR"<br>params:<br>T_max: 20<br>eta_min: 1e-6

Reference

1. Ashish Vaswani NS, Niki Parmar, Jakob Uszkoreit, Llion Jones, Aidan N Gomez, Łukasz Kaiser, Illia Polosukhin. Attention is all you need. *Advances in neural information processing systems*. 2017;(30):5998-6008.
2. Pérez-García F, Sharma H, Bond-Taylor S, et al. Exploring scalable medical image encoders beyond text supervision. *Nature Machine Intelligence*. 2025;7(1)
3. Li YZSYYLR. A Closer Look at the CLS Token for Cross-Domain Few-Shot Learning. *Annual Conference on Neural Information Processing Systems*. 2024;
4. Wang C, Shao J, He Y, et al. Data-driven risk stratification and precision management of pulmonary nodules detected on chest computed tomography. *Nat Med*. 2024;30(11):3184-3195.
5. Altorki N, Wang X, Kozono D, et al. Lobar or Sublobar Resection for Peripheral Stage IA Non-Small-Cell Lung Cancer. *N Engl J Med*. 2023;388(6):489-498.
